# A lightweight machine learning approach for DDoS detection and classification

**DOI:** 10.1038/s41598-026-48535-x

**Published:** 2026-04-10

**Authors:** Osama Ebrahem, Salah Dowaji, Suhel Alhammoud

**Affiliations:** Department of Computer System and Networking, Faculty of Information Engineering, Damascus University, Al-Tabbaleh, Damascus, Syrian Arab Republic

**Keywords:** DDoS attacks, Machine learning, Intrusion detection, Data Balancing, NearMiss, CIC-DDoS2019, Random Forest, Complement Naïve Bayes, K-nearest-neighbour, Logistic regression, Classification and taxonomy, Machine learning

## Abstract

With the increasing complexity of network environments, Distributed Denial of Service (DDoS) reflection and exploitation attacks have become more diverse, necessitating not only detection but also accurate classification. We aim to find novel findings with universally, less complexity and better efficiency to detect and mitigate these attacks. Hence, this study proposes a lightweight and universal machine learning-based approach for multiclass DDoS detection and subcategory classification. The framework employs several models, including Complement Naïve Bayes, k-Nearest Neighbors (kNN), Random Forest (RF), and Logistic Regression—trained on universal and some minimal universal feature subsets. To address class imbalance and reduce data volume, the NearMiss under-sampling method is applied. Evaluations conducted on the CIC-DDoS2019 dataset demonstrate that the Random Forest classifier offers superior performance in terms of memory usage, while kNN with NearMiss yields faster inference times. The findings support the use of kNN in time-constrained environments and RF in memory-constrained scenarios.

## Introduction

A report from Gcore reveals that distributed denial-of-service (DDoS) attacks have increased in volume and magnitude from Q3-Q4 2023 to Q3-Q4 2024. DDoS attacks increased by 56% year-over-year and 17% since H1 2024. The trend of increasing DDoS attacks is likely to continue, driven by the growing Internet of Things (IoT) ecosystem and the adoption of 5G networks. As attack surfaces expand, we’ll see more sophisticated, multi-vector attacks that combine volumetric assaults with application-layer exploits. Continuous innovation in threat detection and mitigation technologies will be essential to stay ahead of these evolving tactics^1^. The researchers in^2^ proposed universal features set to better detect the botnet attacks regardless of the underlying dataset in the context of the Internet of Things. The proposed features set manifested preeminent results for detecting the botnet attacks when tested the trained machine learning models over three different botnet attack datasets: IoT-23, CTU-13 and CICIDS-2017. In this research, the logistic regression (LR) algorithm was used for feature selection because it is simple, fast and less complex as compared to other techniques^3^. The proposed features set in^2^ used to train four commonly used machine learning algorithms (NB, KNN, RF, LR) for detecting the botnet attacks across three different datasets. The experimental results demonstrated that the machine learning algorithms efficiently detected the botnet attacks when trained and tested these algorithms using the proposed universal features set (Pkt Len Mean, Pkt Size Avg, Fwd Pkts/s, Bwd Pkt Len Min, Pkt Len Min, Down/UpRatio) over three different datasets. While researchers in^4^ proposed a new feature reduction approach, which reduces and weights the most important features from universal features set to fit the big data analytics on IoT based cybersecurity systems. The minimal number of features were chosen by using feature selection methods (ANOVA, Variance Threshold, Information Gain, Chi Square) which performed on the IoT-23 dataset. The general approach consists of two ensemble approaches for features selection. The first sub ensemble approach reduced the number of features to only two features and achieved high results (Pkt Len Mean, Pkt Size Avg). The second sub ensemble approach reduced the number of features to only three features and achieved high results (Pkt Len Mean, Bwd Pkt Len Min, Fwd Pkts/s). It is worth noting that this work focused on detecting DDoS attacks in particular.

The primary contributions of this work are threefold:

1. A lightweight, universal ML framework for multiclass DDoS detection and sub‑category classification, employing a universal feature set and its reduced subsets to balance accuracy with computational efficiency.
2. A thorough evaluation of the NearMiss under‑sampling technique, quantifying its substantial acceleration of inference time (up to 27× for k‑NN) and reduction in memory footprint, thereby enabling deployment in time‑sensitive or resource‑constrained environments.
3. Extensive benchmarking on the CIC‑DDoS2019 dataset across four classical ML models, providing clear guidance for model selection: Random Forest for memory‑limited settings and k‑NN with NearMiss for latency‑critical scenarios.

The rest of the paper is structured as follows. Firstly, an account of related work is presented. A highlight of all the ML techniques that are used in this research is provided. Moreover, the details about the CIC-DDoS2019 dataset are given. Furthermore, the proposed approach is explained as well as all the algorithms involved during the experimental processes. A summary of all the experimental processes and discussions is then presented. The last section of this paper is the conclusion.

## Related work

In this section, we highlight studies that used the CIC-DDoS2019 dataset to evaluate their novel methods to detect DDoS attacks in the network traffic.

The researchers in^5^ proposed a novel deep learning-based intrusion detection system, specifically designed for deployment at either the Cloud or Fog level in the IoT environment. The proposed model aims to detect all types of DDoS attacks with their specific subcategory. Their hybrid model combines different types of deep learning models, including Convolutional Neural Networks (CNNs), Long Short-Term Memory (LSTM), Deep Autoencoder, and Deep Neural Networks (DNNs). Their proposed model is made up of two main levels. The first one contains different parallel sub-neural networks trained with specific algorithms. The second level uses the output of the frozen first level combined with the initial data as input. To evaluate their model, they used the CIC-DDoS2019 dataset, which satisfies all the constraints of an intrusion detection dataset. The results obtained demonstrate that their proposed model outperformed various well-known machine learning and deep learning models in terms of the true positive rate, accuracy, false alarm rate, average accuracy, and average detection rate.

The authors in^6^ proposed a hybrid approach named AE-MLP that combines two deep learning-based models for effective DDoS attack detection and classification. The Autoencoder (AE) part of their proposed model provides an effective feature extraction that finds the most relevant feature sets automatically without human intervention (e.g., knowledge of cybersecurity professionals). The Multi-layer Perceptron Network (MLP) part of their proposed model uses the compressed and reduced feature sets produced by the AE as inputs and classifies the attacks into different DDoS attack types to overcome the performance overhead and bias associated with processing large feature sets with noise (i.e., unnecessary feature values). Their experimental results, obtained through comprehensive and extensive experiments on different aspects of performance on the CICDDoS2019 dataset, demonstrate both a very high and robust accuracy rate and F1-score that exceeds 98% which also outperformed the performance of many similar methods. This shows that their proposed model can be used as an effective DDoS defense tool against the growing number of DDoS attacks.

In another related work of research^7^, the authors proposed an intrusion detection system that includes preprocessing procedures and a deep learning model to detect DDoS attacks. For this purpose, various models based on Deep Neural Networks (DNN), Convolutional Neural Networks (CNN), and Long Short Term Memory (LSTM) have been evaluated in terms of detection performance and real-time performance. They tested the suggested model using the CIC-DDoS2019 dataset, which is frequently used in the literature. They applied preprocess techniques such as feature elimination, random subset selection, feature selection, duplication removal, and normalization to the CIC-DDoS2019 dataset. As a result, better recognition performance was obtained for the training and testing evaluations. According to the test results, 99.99% for binary and 99.30% for multiclass accuracy using the CNN-based inception like model gave the best results among the proposed models. In addition, the inference time of the proposed model for various sizes of test data looks promising compared to baseline models with a smaller number of trainable parameters. The proposed IDS system, together with the preprocessing methods, provides better results when compared to state-of-the-art studies.

In^8^, the authors proposed a DDoS mitigation framework for IoT using fog computing to ensure fast and accurate attack detection. The fog provides resources for effective deployment of the mitigation framework, this solves the deficits in resources of the resource-constrained IoT devices. The mitigation framework uses an anomaly-based intrusion detection method and a database. The database stores signatures of previously detected attacks while the anomaly-based detection scheme utilizes k-NN classification algorithm for detecting the DDoS attacks. By using a database containing the attack signatures, attacks can be detected faster when the same type of attack is executed again. The experimental results on the CICDDoS 2019 data set showed that the k-NN classification algorithm proposed for their framework achieves a satisfactory accuracy in detecting DDoS attacks.

Hassan and Vijey^9^ presented a review and analysis of the machine learning-based schemes for securing the SDN environment targeted by DDoS attacks. The schemes’ method, performance metrics, datasets, and other remarks such as benchmarks, strengths, and weaknesses are discussed. The CIC-DDoS 2019 dataset was utilized to evaluate the performance of a set of classification algorithms that are widely used in machine learning-based DDoS attack detection in the SDN environment. The results of the evaluation indicate that XGBoost is able to detect DDoS attacks in SDN with high accuracy, precision, recall, F1-score, and low error.

Ivandro et al.^10^ proposed CyDDoS, an integrated intrusion detection system (IDS) framework, which combines an ensemble of feature engineering algorithms with the deep neural network. The ensemble feature selection is based on five machine learning classifiers used to identify and extract the most relevant features used by the predictive model. This approach improves the model performance by processing only a subset of relevant features while reducing the computation requirement. They evaluated the model performance based on CICDDoS2019. The evaluation considered different validation metrics such as accuracy, precision, F1-Score, and recall to argue the effectiveness of the proposed framework against state-of-the-art IDSs. The results of the evaluation indicate that their proposed model can reduce 89% of features from the CICDDoS2019 dataset and is trained in five seconds. Overall, the experimental results reveal that their proposed design outperforms its competitors, attaining accuracy of 99.6% and the ROC value nearly to 1, based on the test dataset.

According to the research conducted by Jansi et al. in^11^, the research objective of this work is to identify and prevent DDoS and Denial-of-Service (DoS) attacks (i.e., SYN, Slowloris) in a D2D communication environment. Specifically, by replicating a real-world scenario, they emulate SLowloris attacks in a D2D communication network and generate a D2D Network-specific Slowloris dataset. This dataset al.ong with the CICDDoS2019 dataset was then used to train their proposed Machine learning (ML) model that aids in the detection and prevention of DDoS attacks (Slowloris and SYN) in the considered D2D framework. The whole process of how to construct an emulation network for D2D communication and test it against a variety of attacks and implementations is also demonstrated in the paper. To quantify the detection accuracy in the context of DDoS and DoS attacks, they used various ML algorithms such as Random Forest, Light GBM, XGBoost, and AdaBoost and studied their performance with the aid of extensive emulation. The results collected revealed that both Slowloris and CICDDoS2019 datasets achieve greater accuracy with Random Forest.

Ebtihal and Onytra^12^ involved investigating several machine learning models and employing them with the DDoS detection system. This work investigates the issue of enhancing the DDoS attacks detection accuracy using a well known DDoS named as CICDDoS2019 dataset. In addition, the DDoS dataset has been preprocessed using two main approaches to obtain the most relevant features. Four different machine learning models have been selected to work with the DDoS dataset. According to the results obtained from real experiments, the Random Forest machine learning model offered the best detection accuracy with (99.9974%), with an enhancement over the recently developed DDoS detection systems.

Sharmin and Abdullah^13^ proposed a deep learning-based model using a contractive autoencoder to detect anomalies. They trained their model to learn the normal traffic pattern from the compacted representation of the input data, and then apply a stochastic threshold method to detect the attack. Three renowned Intrusion Detection System datasets have been used for evaluationCIC-IDS2017, NSL-KDD, and CIC-DDoS2019. They have assessed the results against a basic autoencoder and other deep learning approaches to show their model efficacy. Their results indicate a successful intrusion detection of the proposed method with an accuracy ranging between 93.41% and 97.58% on the CIC-DDoS2019 dataset. Moreover, it achieved an accuracy of 96.08% and 92.45% on NSL-KDD and CIC-IDS2017 datasets, respectively.

The research^14^ proposed a novel network architecture called DDoS-MSCT, which combines a multiscale convolutional neural network and transformer. The DDoS-MSCT architecture introduces the DDoS-MSCT block, which consists of a local feature extraction module (LFEM) and a global feature extraction module (GFEM). The LFEM employs convolutional kernels of different sizes, accompanied by dilated convolutions, with the aim of enhancing the receptive field and capturing multiscale features simultaneously. On the other hand, the GFEM is utilized to capture long-range dependencies for attending to global features. Furthermore, with the increase in network depth, DDoS-MSCT facilitates the integration of multiscale local and global contextual information of traffic features, thereby improving detection performance. Their experiments are conducted on the CIC-DDoS2019 dataset, and also the CIC-IDS2017 dataset, which is introduced as a supplement to address the issue of sample imbalance. Experimental results on the hybrid dataset showed that DDoS-MSCT achieves accuracy, recall, F1 score, and precision of 99.94%, 99.95%, 99.95%, and 99.97%, respectively. Compared to the state of the art methods, the DDoS-MSCT model achieved a good performance for detecting the DDoS attack to provide the protecting ability for network security.

The researchers in^15^ proposed an innovative approach for DDoS detection by leveraging Convolutional Neural Networks (CNN), adaptive architectures, and transfer learning techniques. To evaluate the performance of their proposed adaptive transfer learning models, they selected four well-known datasets in cyber security: KDDCup99, UNSW-NB15, CSE-CIC-IDS2018 and CICDDoS2019. Experimental results on publicly available datasets show that the proposed adaptive transfer learning method effectively identifies benign and malicious activities and specific attack categories.

In^16^, the researchers proposed a DDoS attack detection method that combines self attention mechanism with CNN-BiLSTM to address the issues of high dimensionality, multiple feature dimensions, low classification task accuracy, and high false positive rate in raw traffic data. Firstly, the random forest algorithm was combined with Pearson correlation analysis to select important features as model inputs to reduce the redundancy of input data. Secondly, one-dimensional convolutional neural networks and bidirectional long-term and short-term memory networks were used to extract spatial and temporal features respectively, and then the extracted features were ‘‘parallelized’’ to obtain fused features. Once again, an attention mechanism was introduced to ensure that useful input information features are fully and completely expressed, and different weights are given based on the importance of different features. Finally, the softmax classifier was used to obtain the classification results. To verify the effectiveness of the proposed method, binary and multi classification experiments were conducted on the CIC-ISD2017 and CICDDoS2019 datasets. The experimental results showed that compared with existing models, the proposed model had the highest accuracy, precision, recall, and F1 values of 95.670%, 95.824%, 95.904%, and 95.864%, respectively.

Qin et al.^17^ designed their algorithm for DDoS detection and classification based on federated learning. Different terminals only need to pass model gradient parameters rather than directly interact with the collected data, which can not only reduce communication costs, but protect privacy as well. Considering that network structure should not be too complex for high-rated traffic classification, they use a simplified residual network with fewer parameters for detection and classification. In order to test the classification effect of their network structure, they trained the lightweight residual neural network under the federated learning architecture on the random data blocks of CICDDoS2019 and obtained the classification accuracy of all kinds of traffic. In addition, they used the existing neural network model with good performance of DDoS detection also trained CICDDoS2019. Adopted models were DCNN and LSTM. They used traditional machine learning architectures for both models, and each used the same structure, data processing methods, optimizers, loss functions, and hyperparameters. Finally, they proved that their method can obtain accurate classification results even in the case of extreme data distribution.

The research^18^ presented a machine learning model that takes the imbalance nature of the DDoS attack data into consideration for both presence/absence and the type of DDoS attacks detection. They used six machine learning techniques to determine their efficacy in detecting DDoS attack and their types, namely, LR, SVM, DT, RF, ANN and KNN. Moreover, they used SMOTE with selected weights for each class in addressing the class imbalance issue in the DDoS dataset. Extensive experiment analysis with the two recent and extensive datasets, namely, CICIDS 2017 and CICDDoS-2019 showed that the proposed technique can effectively identify DDoS attacks.

In^19^, the researchers presented a system of detection and mitigation of Distributed Denial of Service (DDoS) attacks and Portscan attacks in SDN environments (LSTM-FUZZY). The LSTM-FUZZY system presented in this work has three distinct phases: characterization, anomaly detection, and mitigation. The system was tested in two scenarios. In the first scenario, they applied IP flows collected from the SDN Floodlight controllers through emulation on Mininet. On the other hand, in the second scenario, the CICDDoS 2019 dataset was applied. The results showed the efficiency of the system to assist in network management, detect and mitigate the occurrence of the attacks.

In^20^, an artificial neural network (ANN) based hybrid GBS (Grey Wolf Optimizer (GWO) + Backed Propagation Network (BPN) + Self Organizing Map (SOM)) Intrusion Detection System (IDS) was proposed for intrusion detection in the cloud computing environment. The base classifier, BPN, was chosen for their researched after evaluating the performance of a comprehensive set of neural network algorithms on the standard benchmark UNSW-NB 15 dataset. BPN intrusion detection performance was further enhanced by combining it with SOM and GWO. Hybrid feature selection (fs) was made used a correlation-based approached and stratified 10-fold cross-validation (STCV) ranking based on weight matrix valued (W). These selected features were further fine-tuned used metaheuristic GWO hyperparameter tuning based on a fitness function. The proposed ids technique was validated used the standard benchmark UNSW-NB 15 dataset, which consists of 1,75,341 and 82,332 attacked cases in the training and testing datasets. This study’s findings demonstrate that the proposed ANN-based hybrid GBS ids model outperforms other existing ids models with a higher intrusion detection accuracy of 99. 40%, fewer false alarms (0. 00389), less error rate (0. 001), and faster prediction time (0. 29 ns).

In^21^, the researchers were developed IDS models to detect this attack efficiently, based on machine learning algorithms such as C4.5, SVM, and KNN classifier algorithms and 10-fold cross validation techniques. The NSL-KDD bench mark dataset was employed to validate the models experimentally. A 10-fold cross validation technique was used to select the trend features, and ten trial runs were made to avoid biased output. The classic SVM classifier model reported better accuracy, but the precision and sensitivity of the C4.5 classifier algorithm are better than that of SVM and KNN models. In order to improve the performance of the machine learning based intrusion detection models, an attempt was made to feed the SVM and KNN based IDS model with the features selected by C4.5 classifier algorithm, and the obtained performance metric values are reported. It was evident from the results obtained that the hybrid combination of C4.5 with SVM out performed all other models discussed in this research with an accuracy of 0.9604.

In another related work of research^22^, the authors implemented Artificial Neural Network (ANN) algorithms like Backpropogation neural network (BPN) and Multilayer perceptron (MLP) and demonstrated their performance on intrusion detection by utilizing NSL-KDD dataset. Initially, NSL-KDD benchmark dataset construction was carried out in the range of (0–1) using min-max normalization technique. Following this, hybrid Harris Hawks optimization particle swarm optimization (HHO-PSO) was employed to reduce the dataset size by selecting significant features that represents anomaly in network traffic. This hybrid algorithm was also employed to tune the features selected which is assigned as initial weight vectors for both BPN and MLP intrusion detection system (IDS) models. These selected optimally tuned features were trained using 10-fold cross validation technique and the number of hidden neurons is fixed using thumb rule. After training, the hybrid BPN-MLP neural network IDS model was validated on test dataset and its performance is validated using performance metrics such as accuracy, precision, sensitivity, specificity and F1 score. The proposed hybrid HHO-PSO BPN and HHOPSO MLP IDS model had achieved detection accuracy of and with F1 score of 0.9743 and 0.9800 respectively.

The research^23^ presented the results of various experiments carried out using data mining and machine learning algorithms as well as a combination of these algorithms on the commonly available dataset named CAIDA for TCP SYN flood attack detection. Also, this research analyzed the various performance metrics such as false positive rate, precision, recall, F-measure and receiver operating characteristic (ROC) using various machine learning algorithm. One-R(OR) with an ideal FPR value of 0.05 and recall value of 0.95,decision stump(DS) with an ideal precision value of o.93,PART with an excellent F-measure value of 0.91 were some of the performance metric values while performing TCP SYN flood attack detection.

The research^24^ proposed a solution to some of the limitations in the existing IDS models for DDoS attack detection such as delayed convergence, local stagnation issues, and local and global optimal trapping issues. These limitations were met by the recurrent neural network (RNN) and deep learning- (DL-) based proposed models that can utilize the previous states of the hidden neuron. The proposed research had used a long short-term memory (LSTM) recurrent neural network and autoencoder- and decoder-based deep learning strategy with gradient descent learning rule. The network parameters like weight vectors and bias coefficient were tuned optimally by employing the proposed a hybrid Harris Hawks optimization (HHO) and particle swarm optimization (PSO) algorithm. The proposed hybrid optimization algorithm selected the essential attributes, and the results obtained confirmed that the proposed LSTM and deep learning model outperformed all other models developed in the literature.

In^25^, a hybrid ML intrusion detection system (IDS) model was proposed. The performance of the proposed IDS model was improved by employing a 10-fold cross-validation technique to perform feature selection, reducing data dimensions on the publicly available benchmark NSL-KDD dataset. Performance validation of the proposed hybrid IDS model was done using the confusion matrix. Support vector machine (SVM) parameters were fine-tuned using hybrid Harris Hawks optimization (HHO) and particle swarm optimization (PSO) algorithms. The performance of these hybrid algorithms was compared with other classical algorithms such as C4.5, K-nearest neighbor, and SVM using performance metrics such as precision, sensitivity, specificity, F1 score, and accuracy. From these comparisons, it can be inferred that the proposed SVM with hybrid optimization HHO-PSO machine learning IDS model performs better DDoS detection with good performance metric values.

The research^26^ proposed Trident, a low-rate DoS attack mitigation scheme based on port and traffic state in SDN. Specifically, they designed a multi-step strategy to monitor switch states. First, Trident identifies switches suspected of suffering from low-rate DoS attacks through port state detection. Then, it monitors the traffic state of switches with abnormal port states. Once a switch is identified as suffering from an attack, Trident analyzes the flow information to pinpoint the malicious flow. Finally, Trident issues rules to the switch’s flow table to block the malicious flow, effectively mitigating the attack. They prototyped Trident on the Mininet platform and conducted experiments using a real-world topology to evaluate its performance. The experiments showed that Trident can accurately and robustly detect low-rate DoS attacks, respond quickly to mitigate them, and maintain low overhead.

The researchers in^27^ implemented numerous machine learning techniques to detect the attacks. Conventional machine learning models identify the essential features through specific feature extraction techniques which increases the computation complexity of the system. For attack details and their sub-categories, deep learning technique was used in the proposed work. The detection model incorporates ResNet based on Inception with a support vector machine to detect WSN intrusions. Proposed algorithm was applied to Standard NSL-KDD data set and performance metrics like recall, precision, accuracy and f1-score were considered for analysis. The comparative analysis demonstrated the proposed model performance of 99.46% accuracy is better than traditional approaches like random forest, decision tree, deep neural network and convolutional neural network.

The researchers in^28^ proposed a novel evolve-based phishing scams detection method (named GrabPhisher) that extracts temporal features of accounts and captures information about the dynamic topology of the graph as it evolves. Specifically, GrabPhisher can build the evolutionary pattern of accounts trading on Ethereum as a diffusion network graph in continuous time. It can continue to capture new transaction features based on existing transactions, which facilitates the identification of phishing accounts. Additionally, we implement GrabPhisher on the real-world Ethereum phishing scams datasets. Extensive experimental results demonstrated that GrabPhisher can effectively extract dynamic temporal features and outperform state-of-the-art methods (95% Recall, and 88% F1-score).

The research^29^ put forward a novel deep learning vulnerability detection method based on opcode-level analysis, designated as NDLSC. The method initially transformed smart contracts into opcodes, subsequently employed the Skip-Gram model in Word2Vec to vectorise the dataset. Subsequently, the Residual Networks 34(ResNet-34) deep learning model was utilized for feature extraction, followed by the Kolmogorov-Arnold Networks(KAN) model for further feature extraction and classification. This approach was employed with the objective of achieving superior results. The core algorithm of NDLSC, which combines ResNet and KAN, is experimentally compared with existing vulnerability detection techniques. The findings demonstrated that this combination not only enhances the precision of smart contract vulnerability identification but also fortifies the resilience of the model. By organically combining these two structures, the understanding and detection of smart contracts were significantly improved, making the detection process more precise and reliable.

In another related work of research^30^, two level security mechanisms have deployed. In level one, an entropy-based mechanism was proposed to detect the DDoS flooding attack in the early stage by temporarily holding the particular flow. In level two, a machine learning-based C4.5 technique was proposed to detect the attack by analyzing additional features and send a permanent alert to drop the packets. The results were analyzed with K-fold validation technique in terms of sensitivity, specificity and accuracy.

The authors in^31^ proposed a DDoS attack detection method based on abnormal alarm and deep detection. First, they used the anomaly detection capability of interquartile range (IQR) to monitor the packet_in message rate of each switch and design a dynamic threshold alarm algorithm. This algorithm can preliminarily identify abnormal switches. In addition, they proposed an integrated-feature-selection method to expose the most-relevant flow features, and extract new SDN flowtable features. Based on these features, they designed a Deep Feature Fusion Convolutional Neural Network (DFFCNN) model to execute deep DDoS attack detection. This model combines a self-attention mechanism with multi-scale features extraction, enhancing its ability to capture data patterns. Experimental results on three typical datasets—IDS2017, IDS2018, and DDoS2019—demonstrated that the proposed method achieves an average detection accuracy of 99.54% and a false positive rate of 0.53%. This represents an improvement of 1.65% over existing detection methods and reduces the false positive rate by 1.38%. Additionally, the proposed two-stage detection method decreases CPU utilization by an average of 12.8% to the existing polling detection method.

In summary, the related works exhibit shortcoming, to the best of our knowledge, none of these studies try to propose a lightweight machine learning approach based on the universal features set which was proposed in^2^, nor even the minimum universal features sets proposed in^4^. Although they claimed that their features performance that they proposed were not limited to a specific dataset on which they are trained.

## Overview of ML methods

The following section provides a review of the supervised ML approaches that are used in this work. We use four machine learning algorithms, i.e.: NB, k-NN, RF and LR. We chose these algorithms for two main reasons. The first is because they were used in both researches^2,4^. Therefore, the experiments in this paper will serve as an improvement and complement to the experiments in these two papers. The second reason is that we aim to find novel findings with universally, less complexity and better efficiency to detect and mitigate DDoS attacks. In experimenting data classification, the dataset is divided into 70% for training and 30% for testing.

### NAIVE BAYES (NB)

Bayesian classification is a statistical classification that is able to predict the probability of class membership. Bayesian classification is based on the Bayes theorem^32^. The Bayesian classification is better known as the Naïve Bayes classification. Naïve Bayes assumes that the influence of attribute values on class is independent of other attribute values. Some anomaly detection studies using Naive Bayes include research conducted by^33–35^.

### K-Nearest-Neighbour (KNN)

The k-Nearest-Neighbour (kNN) ML algorithm is capable of conducting both supervised and unsupervised processes. For instance, kNN is the basis of many of the clustering algorithms in use today^36^. In this research, we used the kNN method in its supervised ML flavor. This technique bases itself on the Standard Euclidean Metric (EM). The EM is a distance that separates two points (instances) in a space^37^. Lets p and q denote two instances in an Euclidean space Z, the EM between p and q, ∆(p, q), is computed as follows:

1 $$\:\varDelta\:\:(\mathrm{p},\:\mathrm{q})=\sqrt{\sum\:_{\mathrm{i}=1}^{\mathrm{n}}{\left({\mathrm{p}}_{\mathrm{i}}-{\mathrm{q}}_{\mathrm{i}}\right)}^{2}}$$

n denotes the maximum number of instances within the space Z. The kNN technique finds the identity (label) of an example n0 within Z by calculating the EM separating n0 and its k closest instances within Z and the label (class) of n0 is selected with reference to the class of its k related neighbouring instances. Some anomaly detection studies that use k-Nearest-Neighbour include research conducted by^38–40^.

### Random Forest (RF)

Random Forest is one of the ensemble classifier methods. If a classifier in an ensemble is a decision tree classifier, then the collection of classifiers is a ‘‘forest’’. Each decision tree is created through a random selection of attributes at each node for separation^41^. The random forest algorithm was proposed by Breich in 2001^42^. Some anomaly detection studies that use random forest include research conducted by^43–45^.

### Logistic Regression (LR)

Logistic Regression (LR) is a ML technique that is primarily employed for the binary classification task although it is termed as “regression”. The LR can also be used for the multiclass classification tasks whereby the learning algorithm employs the one-vs rest methods. The LR model applies the sigmoid function or its variations to a linear ML model. The output of this operation is squashed between [0, 1]. An output that is closer to 1 determines the probability of a given class. The mathematical formulation is described in Eqs. 2, 3 and 4^46^.

2 $${\text{Linear Model: y}} = {\mathrm{b}} + {\mathrm{w}}_{{\mathrm{1}}} {\mathrm{x}}_{{\mathrm{1}}} + {\mathrm{w}}_{{\mathrm{2}}} {\mathrm{x}}_{{\mathrm{2}}} + \cdots + {\mathrm{w}}_{{\mathrm{n}}} {\mathrm{x}}_{{\mathrm{n}}}$$

3 $$\:\mathrm{S}\mathrm{i}\mathrm{g}\mathrm{m}\mathrm{o}\mathrm{i}\mathrm{d}\:\mathrm{E}\mathrm{x}\mathrm{p}\mathrm{r}\mathrm{e}\mathrm{s}\mathrm{s}\mathrm{i}\mathrm{o}\mathrm{n}:\:{\upsigma\:}\:\left(\mathrm{k}\right)=\frac{1}{1+{\mathrm{e}}^{-\mathrm{k}}}$$

4 $$\:\mathrm{L}\mathrm{o}\mathrm{g}\mathrm{i}\mathrm{s}\mathrm{t}\mathrm{i}\mathrm{c}\:\mathrm{R}\mathrm{e}\mathrm{g}\mathrm{r}\mathrm{e}\mathrm{s}\mathrm{s}\mathrm{i}\mathrm{o}\mathrm{n}:\:{\upsigma\:}\:\left(\mathrm{y}\right)=\frac{1}{1+{\mathrm{e}}^{-\mathrm{y}}}$$

In the above formulation, y is processed by σ. Some anomaly detection studies that use Logistic Regression include research conducted by^11,12^.

### Overview of under-sampling method (NearMiss)

NearMiss is a method that aims to create a balanced class distribution by selecting examples based on the proximity of majority class instances to minority class instances^47,48^. Some modern studies that use NearMiss include research conducted by^49,50^. NearMiss was selected as the under-sampling strategy due to its geometric preservation of the majority class distribution, computational efficiency in high-dimensional spaces, and enhanced support for local-structure-sensitive classifiers. Unlike synthetic oversampling methods, it mitigates overfitting risks while maintaining critical decision boundaries.

### CIC-DDoS2019 dataset

For our experimental processes, we utilize the CIC-DDoS2019 dataset^51^. The dataset contains a large amount of up-to-date realistic DDoS attack samples as well as benign samples. Each record of the dataset contains 88 statistical features (e.g., timestamp, source and destination IP addresses, source and destination port numbers, the protocol used for the attack, and a label for a type of DDoS attack). The training dataset contains a total of 12 different DDoS attacks (i.e., NTP, DNS, LDAP, MSSQL, NetBIOS, SNMP, SSDP, UDP, UDP-Lag WebDDoS, SYN, and TFTP) while only three DDoS attacks are included in the testing dataset (i.e., MSSQL, LDAP and UDP). These DDoS attacks cover two different categories; some belong to Reflection-based and others belong to Exploitation-based.

#### Reflection-based attacks

The attackers of the DDoS attack in this category typically send malicious network packets sent to reflector servers with the source IP address set to target the victim’s IP address so that the victim is overwhelmed to send an enormous number of response packets. These attacks are typically carried out through application layer protocols. In terms of our CICDDoS2019 dataset, any traffic with the (application layer) protocol defined for MSSQL, SSDP, NTP, TFTP, DNS, LDAP, NetBIOS, and SNMP be reflection-based attacks.

#### Exploitation-based attacks

The attackers of the DDoS attack in this category exploit a particular protocol used in the network, transport, and application level of the Open Systems Interconnection (OSI) model or TCP/IP 5-layer model. Transport layer protocols such as TCP or UDP are typically used to overwhelm the victim’s IT resources (e.g., SYN flood, UDP flood, and UDPLag) by sending a massive number of TCP or UDP packets. The dataset labeled with SYN, UDP, and UDP-lag in CIC-DDOS2019 belongs to this category. This dataset is characterized by its realistic background traffic generated using the B-Profile system. The dataset includes labeled flows, making it suitable for training and testing machine learning and deep learning models for DDoS detection.

In our work, we use three DDoS attack types (i.e., MSSQL, LDAP and UDP) and benign traffic samples to train and test our proposed model.

## Proposed approach

**Fig. 1:**
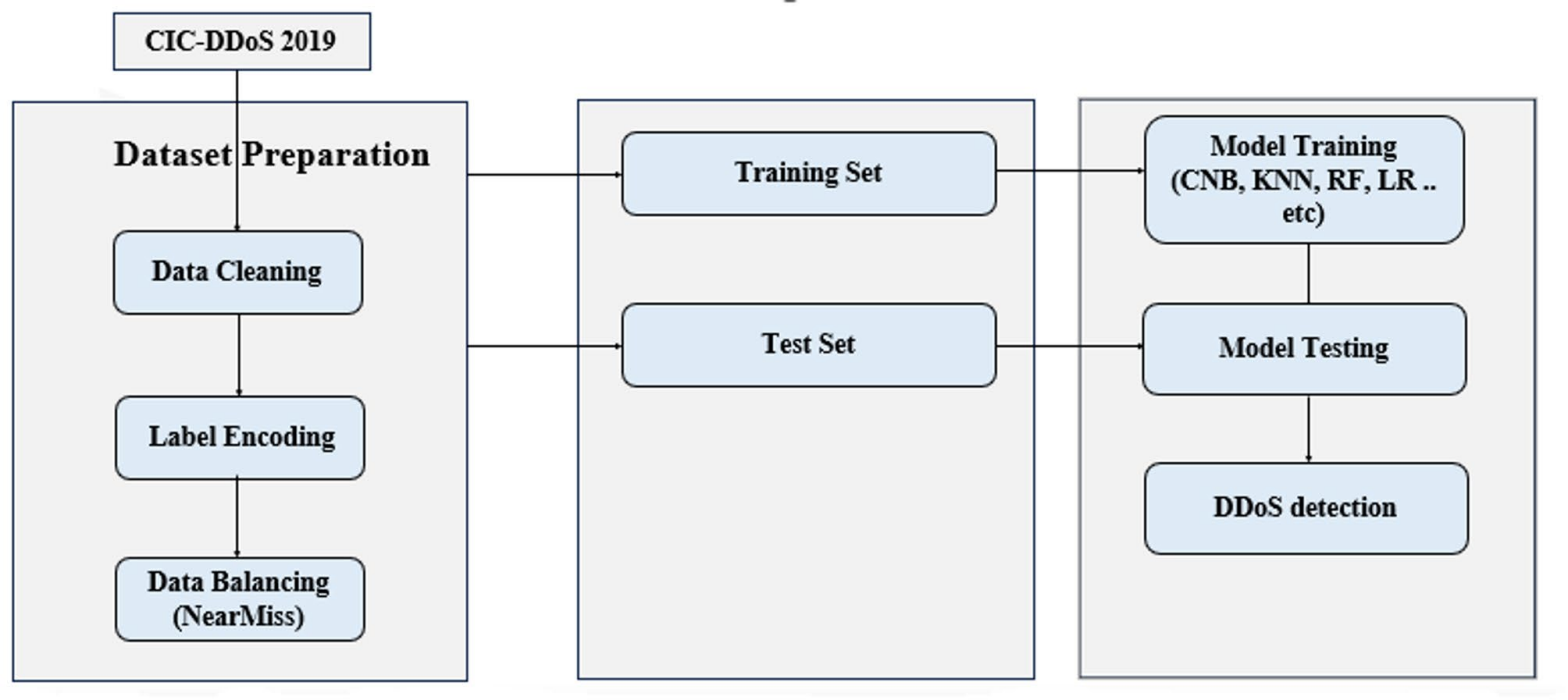
The general architecture of a proposed approach in this paper.

The general architectural design of the approach proposed in this research is detailed in Fig. 1. The first block is all about data preprocessing. This process is often referred to as data engineering. This step is critical for a successful learning process. Data preprocessing has three main steps, namely, data cleaning, label encoding and data balancing. The next block in the middle indicates that the data was divided into 70% for training and 30% for testing. The third block involves model training using the training set. Finally, in the same block, the supervised machine learning classifiers CNB, K-NN, RF and LR are tested with the performance the different features subsets according to two cases: the imbalanced file after data preprocessing and the small balanced file which resulted from applying the under-sampling technique.

## Methodology

### Dataset preparation

In this section, we discuss the stages we used to process our dataset to feed into ML algorithms used in our research. When we downloaded the CIC-DDoS2019 dataset, we noticed that its files were in CSV format and were labeled, so we only needed two main steps when pre-processing the data: data cleaning and Label Encoding. We have provided a link to the source code for reproducibility.

### Data cleaning

The original dataset contained 88 features. Removing certain fields from The CIC-DDoS2019 dataset was our first step in the data cleaning stage. We dropped all metadata features mentioned in^52^ features such as source and destination IP addresses, ports and time-based features because they give the models not real accuracies for the reason that it is possible that included in the training stage lead to overly optimistic results. In addition, we dropped the Flow ID field because it consists of the five tuples, i.e., source IP, source port, destination IP, destination port and protocol. Then we rectified the noisy data, null value data and infinity data values. In the noisy data rectifying step, we have removed duplicate records because it can help reduce noise and redundancy in our dataset. We further have removed rows or columns with a significant amount of missing data. Finally, we have replaced infinity data with missing values and have removed it.

### Label encoding

We had to substitute the categorical labels as ML models only operate on float/numeric values. One categorical value we had to convert was the attack label (i.e., benign and the four attack types). In this stage, we utilized a label encoder from the Sklearn package to transform network traffic statistics. Using this tool, we efficiently encoded string tags and categorical features into numerical values. This transformation assigns integer values ranging from 0 to *n* − 1 to the non-numerical data, where n is the number of distinct categories in each feature. For example, if a feature has three categories (A, B, and C), n would be 3, and the categories might be encoded as 0, 1, and 2 respectively. This encoding makes the data suitable for preprocessing by the ML method. The source code of the process of data cleaning and label encoding are publicly available at link: https://github.com/Eng-Osama-Ebrahem87/A-Lightweight-Machine-Learning-Approach-for-DDoS-Detection-and-Classification/blob/main/Data%20Cleaning%20and%20Feature%20Engineering%20Stages_CIC-DDos%202019.py.

### Data balancing

We handled Imbalanced data problem in CIC-DDoS2019 using NearMiss method:

Under-sampling Method: by keeping all samples in the rare class and randomly selecting an equal number of samples in the abundant class using NearMiss technique. The source code of the process of Under-sampling are publicly available at link: https://github.com/Eng-Osama-Ebrahem87/A-Lightweight-Machine-Learning-Approach-for-DDoS-Detection-and-Classification/blob/main/NearMiss_Under-sampling.py.

## Experiments and results

Our experimental design was categorized in three main stages whereby we considered the following ML techniques: CNB, KNN, RF, and LR. In the first stage of the experiments, we have executed the data preprocessing operations, which has three main steps, namely, data cleaning, label encoding and data balancing. Then in the second stage of the experiments, we have trained and tested ML algorithms according to the universal features set which was proposed in^2^ on the CIC-DDoS2019 Dataset. In the last stage, we have trained and tested ML algorithms according to the minimal universal features subsets which were proposed in^4^ on the CIC-DDoS2019 Dataset. In all the previous stages, we repeated each experiment three times, and then we took the arithmetic average of the time consumed. We have chosen the Complement Naive Bayes classifier from five types of Naive Bayes available in Scikit-Learn library for two reasons, the first is that it is particularly suited for imbalanced data sets and the second is because it is used in^4^. The source codes of the four ML algorithms are publicly available at link: https://github.com/Eng-Osama-Ebrahem87/A-Lightweight-Machine-Learning-Approach-for-DDoS-Detection-and-Classification.

### Hardware and environment setting

The experiments presented in this work are conducted on a HP loaded with the Windows 10 Operating System with the following processor: Intel(R) Core(TM) i7-10510U CPU @ 1.80 GHz to 2.30 GHz. The installed RAM was 16.0 GB. The ML models are built, trained, evaluated and tested on the Scikit-Learn ML Python framework^53^. Scikit-Learn is a versatile open source platform that is constructed on top of matplotlib, NumPy and Scipy Python libraries. Moreover, Classification, Regression and Clustering tasks can all be conducted using Scikit-Learn.

### Performance metrics

There exist a number of metrics to evaluate ML based IDS systems; however, this research aims to maximize the correct predictions of instances in the test dataset. The main measure to look at is the F1-score because we test our ML algorithms on an imbalanced dataset (CIC-DDoS2019). The F1-score metric defined below:

5 $$\:\mathrm{F}1-\mathrm{s}\mathrm{c}\mathrm{o}\mathrm{r}\mathrm{e}=2\frac{\mathrm{P}\mathrm{r}\mathrm{e}\mathrm{c}\mathrm{i}\mathrm{s}\mathrm{i}\mathrm{o}\mathrm{n}.\mathrm{R}\mathrm{e}\mathrm{c}\mathrm{a}\mathrm{l}\mathrm{l}}{\mathrm{P}\mathrm{r}\mathrm{e}\mathrm{c}\mathrm{i}\mathrm{s}\mathrm{i}\mathrm{o}\mathrm{n}\:+\:\mathrm{R}\mathrm{e}\mathrm{c}\mathrm{a}\mathrm{l}\mathrm{l}}$$

Additional main metrics we consider in this paper are the Recall and the Precision that defined below:

6 $$\:\mathrm{P}\mathrm{r}\mathrm{e}\mathrm{c}\mathrm{i}\mathrm{s}\mathrm{i}\mathrm{o}\mathrm{n}=\frac{\mathrm{T}\mathrm{P}}{\mathrm{T}\mathrm{P}\:+\:\mathrm{F}\mathrm{P}}$$

7 $$\:\mathrm{R}\mathrm{e}\mathrm{c}\mathrm{a}\mathrm{l}\mathrm{l}=\frac{\mathrm{T}\mathrm{P}}{\mathrm{T}\mathrm{P}\:+\:\mathrm{F}\mathrm{N}}$$

Whereby the TP stands for True Positive and is the rate of examples correctly identified as attacks. TN, True Negative, is the rate of legitimate traffic classified as legitimate. FP, False Positive, sometimes referred to as Type I error, is the rate of legitimate traffic classified as attacks. FN, sometimes referred to as Type II error, is the rate of legitimate traffic classified as intrusions.

The F1-score is defined as the harmonic mean of the expression in (6) and (7). Moreover, some applications are designed to be Precision or Recall oriented. In order to achieve that in the training process, the Fβ defined below can be tuned:

8 $$\:\mathrm{F}{\upbeta\:}=\frac{\left(1\:+\:{{\upbeta\:}}^{2}\right)\mathrm{T}\mathrm{P}}{\left(1\:+\:{{\upbeta\:}}^{2}\right)\mathrm{T}\mathrm{P}\:+\:{\upbeta\:}\mathrm{F}\mathrm{N}\:+\:\mathrm{F}\mathrm{P}}$$

Where the parameter β can be adjusted based on the application. In our research however, we use the F1-score, which is a trade-off between the Precision and the Recall.

Additional secondary metric we consider in this paper is the Accuracy (AC) that defined below:

9 $$\:\mathrm{A}\mathrm{c}\mathrm{c}\mathrm{u}\mathrm{r}\mathrm{a}\mathrm{c}\mathrm{y}=\frac{\mathrm{T}\mathrm{N}\:+\:\mathrm{T}\mathrm{P}}{\mathrm{F}\mathrm{P}\:+\:\mathrm{F}\mathrm{N}\:+\:\mathrm{T}\mathrm{P}\:+\:\mathrm{T}\mathrm{N}}$$

It must be noted here that applying Accuracy criterion is more feasible with balanced samples.

**False**-**positive rate** (**FPR**): Machine learning models subsets are evaluated by means of an accuracy performance metric called False positive rate(FPR).In this accuracy measurement is done by making comparisons between the ground truth and the models output.In the case of supervised learning the underlying data is defined as well as described by the ground truth.FPR also termed as fall-out are the negative cases which are wrongly identified as positive cases. Positive class incorrect prediction is termed as false positive (FP)^54^. Negative class correct prediction is termed as True negative (TN). False positive rate (FPR) is the FP value divided by the summation of FP and TN. This metric helps in DDoS attacks.

**Receiver operating characteristic** (**ROC**): ROC curve is a graphical chart for analyzing the capacity of the data mining algorithm. This curve is derived when TPR is plotted against FPR. This curve helps in the optimal selection of algorithms for DDoS detection. If we have a greater area under the curve for a particular algorithm it means it performs better in attack detection.

**Confusion matrix** : It is a simple table used to measure how well a classification model is performing. It compares the predictions made by the model with the actual results and shows where the model was right or wrong. This helps understand where the model is making mistakes so you can improve it. It breaks down the predictions into four categories:

True Positive (TP): The model correctly predicted a positive outcome i.e. the actual outcome was positive.

True Negative (TN): The model correctly predicted a negative outcome i.e. the actual outcome was negative.

False Positive (FP): The model incorrectly predicted a positive outcome i.e. the actual outcome was negative. It is also known as a Type I error.

False Negative (FN): The model incorrectly predicted a negative outcome i.e. the actual outcome was positive. It is also known as a Type II error^55^.

### Classification results

The following experimental results help in the study of the effects of subsets of universal features set on the performance of ML Algorithms (CNB, KNN, RF, and LR) on the CIC-DDoS2019 dataset. This results clearly exhibits how each classifier behaves with different subsets of universal features set. We have indicated class distribution very precisely in the header of each of the results tables. The source code link also indicates the files on which the experiments were performed. All of the above facilitates the reproduction process. As for the hyperparameter settings, we did not adjust the value of these parameters, but rather kept the default settings as in the two researches^2,4^. In our experiments, we focused on feature sets that have been proposed in^2^ and reduced in^4^. The results of our experimental processes are listed in Tables 1, 2, 3, 4, 5, 6, 7, 8, 9, 10, 11, 12, 13, 14, 15 and 16 as follows:

**Table 1:** The results of DDOS attack detection on CIC-DDoS2019 for universal features set (Packet Length Mean, Average Packet Size, Bwd Packet Length Min, Fwd Packets/s, Min Packet Length, Down/Up Ratio), Sample Size is: 1120.76 MB, analyze class distribution Label: [Benign: 2789, LDAP: 9931, MSSQL: 5706080].

Classifier	Label	Accuracy	Precision	Recall	F1-score	Training time (S)	Prediction time (S)	Memory usage (B)
CNB	Benign	78	99	18	31	0.624	0.078	1768
	LDAP	78	1	99	1			
	MSSQL	78	100	78	87			
KNN	Benign	100	95	93	94	14.918	1316.5	183,919,472
	LDAP	100	74	7	13			
	MSSQL	100	100	100	100			
RF	Benign	100	98	97	98	590.715	13.011	2676
	LDAP	100	95	7	14			
	MSSQL	100	100	100	100			
LR	Benign	100	88	95	91	60.366	0.109	1785
	LDAP	100	13	0	0			
	MSSQL	100	100	100	100			

**Table 2:** The results of DDOS attack detection on CIC-DDoS2019 for universal features set (Packet Length Mean, Average Packet Size, Bwd Packet Length Min, Fwd Packets/s, Min Packet Length, Down/Up Ratio), Sample Size is: 1.66 MB, analyze class distribution Label: [Benign: 2789, LDAP: 2789, MSSQL: 2789], Balanced: NearMiss.

Classifier	Label	Accuracy	Precision	Recall	F1-score	Training time (S)	Prediction time (S)	Memory usage (B)
CNB	Benign	64	57	91	70	0.015	0.0	1768
	LDAP	64	72	99	83			
	MSSQL	64	0	0	0			
KNN	Benign	99	98	100	99	0.0	0.064	271,904
	LDAP	99	100	99	99			
	MSSQL	99	100	99	99			
RF	Benign	99	99	100	99	0.249	0.022	2676
	LDAP	99	100	99	100			
	MSSQL	99	100	99	99			
LR	Benign	99	98	99	98	0.116	0.0	1785
	LDAP	99	99	99	99			
	MSSQL	99	99	98	99			

**Table 3:** The results of DDOS attack detection on CIC-DDoS2019 for universal features subset (Packet Length Mean, Average Packet Size, Bwd Packet Length Min, Fwd Packets/s), Sample Size is: 1120.76 MB, analyze class distribution Label: [Benign: 2789, LDAP: 9931, MSSQL: 5706080].

Classifier	Label	Accuracy	Precision	Recall	F1-score	Training time (S)	Prediction time (S)	Memory usage (B)
CNB	Benign	74	100	19	32	0.587	0.067	1640
	LDAP	74	1	99	1			
	MSSQL	74	100	74	85			
KNN	Benign	100	94	91	93	9.966	1005.9	122,613,920
	LDAP	100	73	7	13			
	MSSQL	100	100	100	100			
RF	Benign	100	97	95	96	673.086	15.116	2660
	LDAP	100	95	7	14			
	MSSQL	100	100	100	100			
LR	Benign	100	85	86	86	63.422	0.098	1769
	LDAP	100	0	0	0			
	MSSQL	100	100	100	100			

**Table 4:** The results of DDOS attack detection on CIC-DDoS2019 for universal features subset (Packet Length Mean, Average Packet Size, Bwd Packet Length Min, Fwd Packets/s), Sample Size is: 1.66 MB, analyze class distribution Label: [Benign: 2789, LDAP: 2789, MSSQL: 2789], Balanced: NearMiss.

Classifier	Label	Accuracy	Precision	Recall	F1-score	Training time (S)	Prediction time (S)	Memory usage (B)
CNB	Benign	63	67	90	77	0.015	0.0	1640
	LDAP	63	60	99	75			
	MSSQL	63	0	0	0			
KNN	Benign	99	98	100	99	0.005	0.051	182,208
	LDAP	99	100	99	99			
	MSSQL	99	100	99	99			
RF	Benign	99	98	100	99	0.286	0.022	2660
	LDAP	99	100	99	99			
	MSSQL	99	100	99	99			
LR	Benign	98	98	99	98	0.123	0.0	1769
	LDAP	98	99	97	98			
	MSSQL	98	98	98	98			

**Table 5:** The results of DDOS attack detection on CIC-DDoS2019 for universal features subset (Packet Length Mean, Bwd Packet Length Min, Fwd Packets/s), Sample Size is: 1120.76 MB, analyze class distribution Label: [Benign: 2789, LDAP: 9931, MSSQL: 5706080].

Classifier	Label	Accuracy	Precision	Recall	F1-score	Training time (S)	Prediction time (S)	Memory usage (B)
CNB	Benign	77	100	23	38	0.597	0.062	1576
	LDAP	77	1	99	1			
	MSSQL	77	100	77	87			
KNN	Benign	100	94	90	92	8.099	928.21	91,961,144
	LDAP	100	73	7	12			
	MSSQL	100	100	100	100			
RF	Benign	100	96	93	95	554.577	14.855	2652
	LDAP	100	95	7	14			
	MSSQL	100	100	100	100			
LR	Benign	100	88	67	76	49.136	0.084	1761
	LDAP	100	0	0	0			
	MSSQL	100	100	100	100			

**Table 6:** The results of DDOS attack detection on CIC-DDoS2019 for universal features subset (Packet Length Mean, Bwd Packet Length Min, Fwd Packets/s), Sample Size is: 1.66 MB, analyze class distribution Label: [Benign: 2789, LDAP: 2789, MSSQL: 2789], Balanced: NearMiss.

Classifier	Label	Accuracy	Precision	Recall	F1-score	Training time (S)	Prediction time (S)	Memory usage (B)
CNB	Benign	64	65	91	76	0.005	0.0	1576
	LDAP	64	62	99	76			
	MSSQL	64	0	0	0			
KNN	Benign	99	98	100	99	0.007	0.068	137,360
	LDAP	99	100	99	99			
	MSSQL	99	100	99	99			
RF	Benign	99	98	99	99	0.228	0.015	2652
	LDAP	99	100	99	99			
	MSSQL	99	100	99	99			
LR	Benign	98	98	97	97	0.104	0.0	1761
	LDAP	98	97	97	97			
	MSSQL	98	98	98	98			

**Table 7:** The results of DDOS attack detection on CIC-DDoS2019 for minimal universal features subset (Packet Length Mean, Average Packet Size), Sample Size is: 1120.76 MB, analyze class distribution Label: [Benign: 2789, LDAP: 9931, MSSQL: 5706080].

Classifier	Label	Accuracy	Precision	Recall	F1-score	Training time (S)	Prediction time (S)	Memory usage (B)
CNB	Benign	88	80	26	39	0.582	0.067	1512
	LDAP	88	1	99	3			
	MSSQL	88	100	88	94			
KNN	Benign	100	89	97	93	6.295	1386.73	61,308,368
	LDAP	100	99	7	13			
	MSSQL	100	100	100	100			
RF	Benign	100	91	98	94	656.304	13.843	2644
	LDAP	100	97	7	13			
	MSSQL	100	100	100	100			
LR	Benign	100	80	55	65	54.307	0.088	1753
	LDAP	100	0	0	0			
	MSSQL	100	100	100	100			

**Table 8:** The results of DDOS attack detection on CIC-DDoS2019 for minimal universal features subset (Packet Length Mean, Average Packet Size), Sample Size is: 1.66 MB, analyze class distribution Label: [Benign: 2789, LDAP: 2789, MSSQL: 2789], Balanced: NearMiss.

Classifier	Label	Accuracy	Precision	Recall	F1-score	Training time (S)	Prediction time (S)	Memory usage (B)
CNB	Benign	61	97	85	90	0.005	0.0	1512
	LDAP	61	0	0	0			
	MSSQL	61	46	99	63			
KNN	Benign	99	98	100	99	0.005	0.058	92,512
	LDAP	99	99	99	99			
	MSSQL	99	100	98	99			
RF	Benign	99	98	100	99	0.232	0.024	2644
	LDAP	99	99	99	99			
	MSSQL	99	100	98	99			
LR	Benign	98	98	100	99	0.067	0.0	1753
	LDAP	98	100	97	99			
	MSSQL	98	98	98	98			

**Table 9:** The results of DDOS attack detection on CIC-DDoS2019 for universal features set (Packet Length Mean, Average Packet Size, Bwd Packet Length Min, Fwd Packets/s, Min Packet Length, Down/Up Ratio), Sample Size is: 747.7 MB, analyze class distribution Label: [Benign: 3119, MSSQL: 24392, UDP: 3360626].

Classifier	Label	Accuracy	Precision	Recall	F1-score	Training time (S)	Prediction time (S)	Memory usage (B)
CNB	Benign	95	3	85	6	0.426	0.056	1768
	MSSQL	95	18	68	29			
	UDP	95	100	96	98			
KNN	Benign	100	95	95	95	10.403	2118.55	108,965,312
	MSSQL	100	87	83	85			
	UDP	100	100	100	100			
RF	Benign	100	98	100	99	207.324	7.617	2676
	MSSQL	100	90	84	87			
	UDP	100	100	100	100			
LR	Benign	99	92	69	79	32.914	0.056	1785
	MSSQL	99	68	32	44			
	UDP	99	99	100	100			

**Table 10:** The results of DDOS attack detection on CIC-DDoS2019 for universal features set (Packet Length Mean, Average Packet Size, Bwd Packet Length Min, Fwd Packets/s, Min Packet Length, Down/Up Ratio), Sample Size is: 1.932 MB, analyze class distribution Label: [Benign: 3119, MSSQL: 3119, UDP: 3119], Balanced: NearMiss.

Classifier	Label	Accuracy	Precision	Recall	F1-score	Training time (S)	Prediction time (S)	Memory usage (B)
CNB	Benign	61	97	81	88	0.0	0.0	1768
	MSSQL	61	47	99	64			
	UDP	61	0	0	0			
KNN	Benign	97	99	99	99	0.01	0.067	303,776
	MSSQL	97	96	97	97			
	UDP	97	97	96	97			
RF	Benign	98	99	100	100	0.296	0.024	2676
	MSSQL	98	98	96	97			
	UDP	98	97	97	97			
LR	Benign	81	96	90	93	0.109	0.0	1785
	MSSQL	81	85	65	74			
	UDP	81	67	89	76			

**Table 11:** The results of DDOS attack detection on CIC-DDoS2019 for universal features subset (Packet Length Mean, Average Packet Size, Bwd Packet Length Min, Fwd Packets/s), Sample Size is: 747.7 MB, analyze class distribution Label: [Benign: 3119, MSSQL: 24392, UDP: 3360626].

Classifier	Label	Accuracy	Precision	Recall	F1-score	Training time (S)	Prediction time (S)	Memory usage (B)
CNB	Benign	56	0	85	0	0.353	0.036	1640
	MSSQL	56	7	19	11			
	UDP	56	100	56	72			
KNN	Benign	100	94	95	95	5.439	1495.74	72,644,480
	MSSQL	100	88	82	85			
	UDP	100	100	100	100			
RF	Benign	100	98	98	98	344.194	8.846	2660
	MSSQL	100	90	85	87			
	UDP	100	100	100	100			
LR	Benign	99	92	70	80	21.473	0.051	1769
	MSSQL	99	0	0	0			
	UDP	99	99	100	100			

**Table 12:** The results of DDOS attack detection on CIC-DDoS2019 for universal features subset (Packet Length Mean, Average Packet Size, Bwd Packet Length Min, Fwd Packets/s), Sample Size is: 1.932 MB, analyze class distribution Label: [Benign: 3119, MSSQL: 3119, UDP: 3119], Balanced: NearMiss.

Classifier	Label	Accuracy	Precision	Recall	F1-score	Training time (S)	Prediction time (S)	Memory usage (B)
CNB	Benign	61	97	81	88	0.0	0.0	1640
	MSSQL	61	47	99	64			
	UDP	61	0	0	0			
KNN	Benign	97	99	99	99	0.0	0.070	203,456
	MSSQL	97	96	97	97			
	UDP	97	97	96	97			
RF	Benign	98	99	100	99	0.327	0.021	2660
	MSSQL	98	97	96	97			
	UDP	98	96	97	97			
LR	Benign	78	97	83	89	0.124	0.0	1769
	MSSQL	78	76	63	68			
	UDP	78	66	89	76			

**Table 13:** The results of DDOS attack detection on CIC-DDoS2019 for universal features subset (Packet Length Mean, Bwd Packet Length Min, Fwd Packets/s), Sample Size is: 747.7 MB, analyze class distribution Label: [Benign: 3119, MSSQL: 24392, UDP: 3360626].

Classifier	Label	Accuracy	Precision	Recall	F1-score	Training time (S)	Prediction time (S)	Memory usage (B)
CNB	Benign	99	12	83	21	0.338	0.031	1576
	MSSQL	99	0	0	0			
	UDP	99	99	99	99			
KNN	Benign	100	95	94	94	4.616	1283.53	54,484,064
	MSSQL	100	90	81	85			
	UDP	100	100	100	100			
RF	Benign	100	96	97	97	270.207	8.875	2652
	MSSQL	100	90	84	87			
	UDP	100	100	100	100			
LR	Benign	99	92	70	80	14.465	0.046	1761
	MSSQL	99	0	0	0			
	UDP	99	99	100	100			

**Table 14:** The results of DDOS attack detection on CIC-DDoS2019 for universal features subset (Packet Length Mean, Bwd Packet Length Min, Fwd Packets/s), Sample Size is: 1.932 MB, analyze class distribution Label: [Benign: 3119, MSSQL: 3119, UDP: 3119], Balanced: NearMiss.

Classifier	Label	Accuracy	Precision	Recall	F1-score	Training time (S)	Prediction time (S)	Memory usage (B)
CNB	Benign	61	97	82	89	0.0	0.0	1576
	MSSQL	61	47	99	64			
	UDP	61	0	0	0			
KNN	Benign	97	99	99	99	0.01	0.062	153,296
	MSSQL	97	95	97	96			
	UDP	97	97	95	96			
RF	Benign	98	99	99	99	0.285	0.031	2652
	MSSQL	98	97	96	97			
	UDP	98	97	97	97			
LR	Benign	79	98	85	91	0.053	0.0	1761
	MSSQL	79	74	64	69			
	UDP	79	69	89	78			

**Table 15:** The results of DDOS attack detection on CIC-DDoS2019 for minimal universal features subset (Packet Length Mean, Average Packet Size), Sample Size is: 747.7 MB, analyze class distribution Label: [Benign: 3119, MSSQL: 24392, UDP: 3360626].

Classifier	Label	Accuracy	Precision	Recall	F1-score	Training time (S)	Prediction time (S)	Memory usage (B)
CNB	Benign	76	86	17	28	0.332	0.032	1512
	MSSQL	76	2	71	4			
	UDP	76	100	76	86			
KNN	Benign	100	97	80	88	2.346	4574.42	36,323,648
	MSSQL	100	85	84	84			
	UDP	100	100	100	100			
RF	Benign	100	96	97	96	224.364	8.441	2644
	MSSQL	100	88	85	86			
	UDP	100	100	100	100			
LR	Benign	99	91	59	71	25.848	0.046	1753
	MSSQL	99	97	23	37			
	UDP	99	99	100	100			

**Table 16:** The results of DDOS attack detection on CIC-DDoS2019 for minimal universal features subset (Packet Length Mean, Average Packet Size), Sample Size is: 1.932 MB, analyze class distribution Label: [Benign: 3119, MSSQL: 3119, UDP: 3119], Balanced: NearMiss.

Classifier	Label	Accuracy	Precision	Recall	F1-score	Training time (S)	Prediction time (S)	Memory usage (B)
CNB	Benign	54	96	15	25	0.0	0.0	1512
	MSSQL	54	62	59	61			
	UDP	54	46	89	60			
KNN	Benign	97	98	100	99	0.01	0.062	103,136
	MSSQL	97	96	96	96			
	UDP	97	97	95	96			
RF	Benign	97	98	100	99	0.278	0.031	2644
	MSSQL	97	96	96	96			
	UDP	97	97	95	96			
LR	Benign	83	97	99	98	0.041	0.0	1753
	MSSQL	83	85	61	71			
	UDP	83	70	89	78			

## Discussions

Our experimental design was implemented according to two distinct cases whereby we considered the following ML techniques: CNB, KNN, RF and LR. In the first case of the experiments, we employed the original imbalanced samples provided by the CIC-DDoS2019 dataset. In the second case, we employed the balanced samples produced using NearMiss technique. Subsequently, we repeated these steps on the features subsets in turn.

With regards to the universal features set which was proposed in^2^, upon reviewing Tables 1, 2, 9 and 10, we found that the Random Forest algorithm often achieved the best performance metrics, where the lowest value of the performance criteria was 96 in the balanced sample using the NearMiss method (see Table 10). The KNN algorithm came in second place, with performance values close to those achieved by the Random Forest algorithm when applied, but it consumed a large size of memory and longtime compared to the other algorithms. However, by applying the NearMiss technique, we observed that KNN outperformed RF in terms of execution time (assuming that the execution time equals the sum of the training time and the prediction time), and we observed a clear decrease in memory usage for the KNN algorithm. Overall, we observed a clear improvement in the performance metrics and a clear decrease in execution time for all algorithms.

For the features subset which consist of four features (Packet Length Mean, Average Packet Size, Bwd Packet Length Min, Fwd Packets/s), upon reviewing Tables 3, 4, 11 and 12, we found also that the Random Forest algorithm often achieved the best performance metrics, where the lowest value of the performance criteria was 96 in the balanced sample using the NearMiss method (see Table 12). The KNN algorithm came in second place, with performance values close to those achieved by the Random Forest algorithm when applied, but it consumed a large size of memory and longtime compared to the other algorithms. However, by applying the NearMiss technique, we observed that KNN outperformed RF in terms of execution time (assuming that the execution time equals the sum of the training time and the prediction time), and we observed a clear decrease in memory usage for the KNN algorithm. Overall, we observed a clear improvement in the performance metrics and a clear decrease in execution time for all algorithms.

About the features subset which consist of three features (Packet Length Mean, Bwd Packet Length Min, Fwd Packets/s), upon reviewing Tables 5, 6, 13 and 14, we found that the Random Forest algorithm often achieved the best performance metrics, where the lowest value of the performance criteria was 96 in the balanced sample using the NearMiss method (see Table 14). The KNN algorithm came in second place, with performance values close to those achieved by the Random Forest algorithm when applied, but it consumed a large size of memory and longtime compared to the other algorithms. However, by applying the NearMiss technique, we observed that KNN outperformed RF in terms of execution time (assuming that the execution time equals the sum of the training time and the prediction time), and we observed a clear decrease in memory usage for the KNN algorithm. Overall, we observed a clear improvement in the performance metrics and a clear decrease in execution time for all algorithms.

In the case of the minimal universal features subset which consist of only two features (Packet Length Mean, Average Packet Size), upon reviewing Tables 7, 8, 15 and 16, we found that the Random Forest algorithm often achieved the best performance metrics, where the lowest value of the performance criteria was 95 in the balanced sample using the NearMiss method (see Table 16). The KNN algorithm came in second place, with performance values close to those achieved by the Random Forest algorithm when applied, but it consumed a large size of memory and longtime compared to the other algorithms. However, by applying the NearMiss technique, we observed that KNN outperformed RF in terms of execution time (assuming that the execution time equals the sum of the training time and the prediction time), and we observed a clear decrease in memory usage for the KNN algorithm. Overall, we observed a clear improvement in the performance metrics and a clear decrease in execution time for all algorithms.

In general, we found that RF achieved the best performance if it compared with other machine learning models. Although the KNN algorithm performed at or near par with RF in many experiments, it consistently suffered from significant prediction time and large memory usage, a result consistent with the results in practice^4^. We also found that universal features subsets achieved somewhat similar values ​​for the performance criteria.

**Fig. 2:**
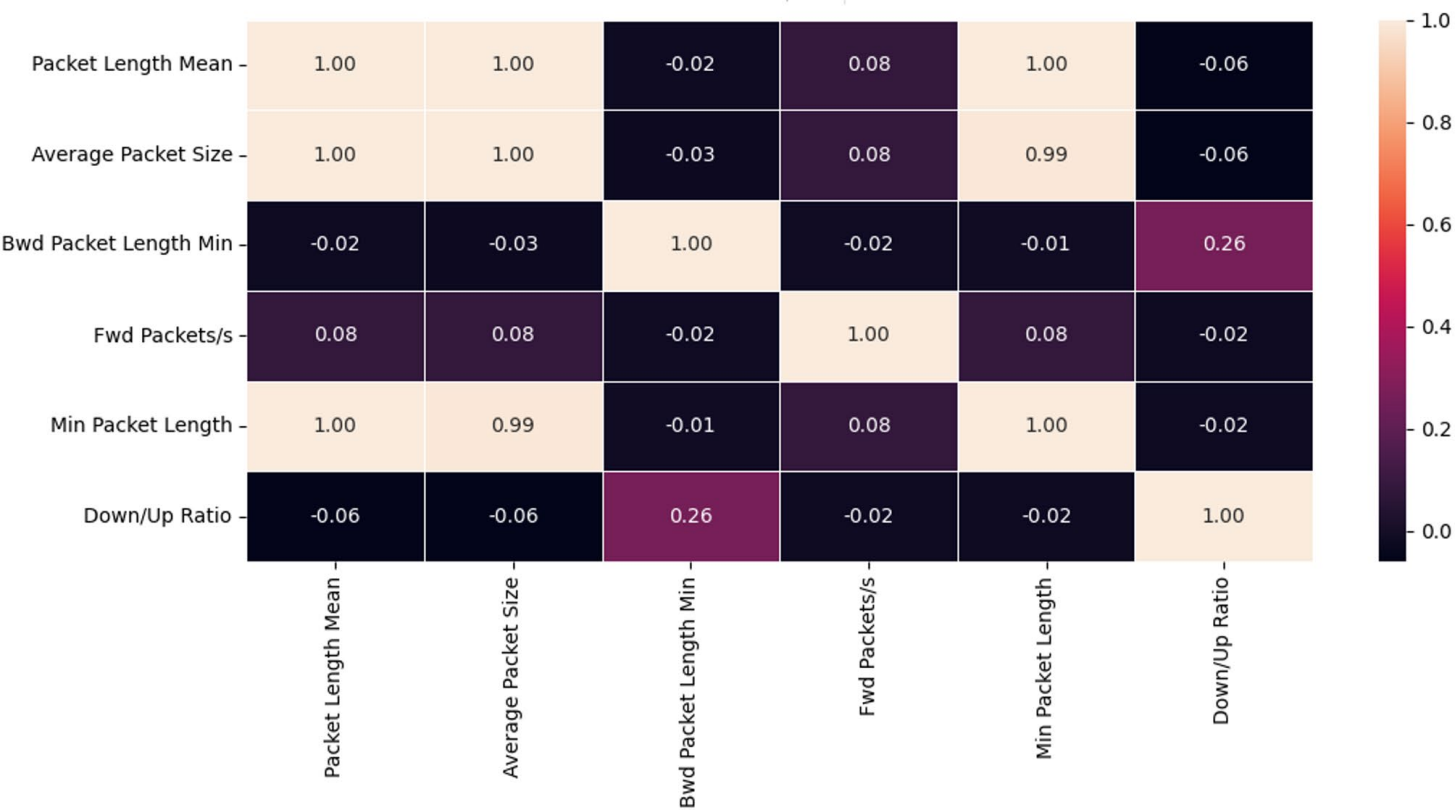
The Pearson correlations matrix for CIC-DDos 2019.

In order to explain the results achieved by machine learning models, we conducted a comprehensive study of the simple correlations between the six features using the Pearson correlation matrix in the context of the CIC-DDos 2019 data set. The results were as shown in the Fig. 2.

The Fig. 2 shows a perfect correlation between the two features “Packet Length Mean” and “Average Packet Size”. We also notice from the Fig. 2a perfect correlation between the two features “Packet Length Mean” and “Min Packet Length”. In addition, we notice a strong correlation between the two features “Average Packet Size” and “Min Packet Length”. As we notice from the Fig. 2 to the existence of varying weak correlations between the rest of the features.

The NB classifier is based upon the Bayes’ theorem with an assumption that all the features used for training the classifier are not correlated. While in our case, the features like “Packet Length Mean” and “Average Packet Size” are correlated with each other. Due to the existence of correlation among features, the Naive Bayes algorithm performed poorly as compared to other algorithms. In addition, The LR classifier is based upon an assumption that the predictor features used for training the classifier should be independent of each other. Therefore, LR classifier can have poor performance if there are highly correlated input variables in a data set. On the other hand, Random forests don’t suffer from correlated variables like linear regression models do. Random forests randomly pick from a subset of variables at each split (hence the “random” in “random forests”). This means that correlated variables are less likely to show up together when the trees are being trained. But even when correlated variables show up in the same random subset of variables, it’s still not much of an issue because the variables aren’t assigned coefficients. This justifies the high performance of the Random Forest algorithm. Finally, it is known that KNN suffers from some limitations and challenges, such as computational expense, slow speed and memory. We have clearly observed this in the experimental results, especially with regard to memory usage.

Finally, we found that KNN with NearMiss achieved better time than RF with NearMiss, but RF still outperformed KNN in terms of memory usage. Therefore, we recommend using KNN with NearMiss when time is a limitation for this network. We also recommend using RF with NearMiss when memory usage is limited in the network environment in which this approach will be applied.

The experimental results demonstrated that the traditional machine learning models achieved commendable performance in detecting known DDoS attack categories, confirming their efficacy within a closed-world assumption. However, when evaluated against previously unseen (out-of-distribution) attacks, a fundamental limitation was exposed: all models failed to reliably identify these novel threats, frequently misclassifying them as benign traffic. This failure is attributed to the intrinsic design of these algorithms, which are optimized to discriminate between a fixed set of predefined classes. They lack an inherent mechanism to recognize samples that deviate significantly from the trained distribution. This finding underscores a critical shortcoming and highlights the imperative to adopt more robust paradigms, such as Open-Set Recognition (OSR) and Anomaly-Based Detection, to effectively counter evolving and zero-day cyber threats. The source code of the experimental stage are publicly available at link: https://github.com/Eng-Osama-Ebrahem87/A-Lightweight-Machine-Learning-Approach-for-DDoS-Detection-and-Classification/blob/main/UnKnown%20DDoS%20Attacks%20Detection_V5_2.py.

Let’s discuss in depth the Implications of High Linear Correlation Between “Packet Length Mean” and “Average Packet Size”:

In the feature engineering phase of constructing machine learning models for Network Traffic Analysis (NTA) and Intrusion Detection Systems (IDS), the selection of informative and non-redundant features is paramount. A strong linear correlation between features such as “Packet Length Mean” and “Average Packet Size” presents significant challenges that can detrimentally impact model integrity. While both features are intuitively valuable for characterizing traffic flows, their collinearity induces several negative effects and limitations. It is as follows:

**Multicollinearity and Model Instability**: High collinearity violates the assumption of feature independence in many linear models (e.g., Logistic Regression, Linear SVM). This leads to instability in the model’s coefficient estimates. Small perturbations in the training data, such as the removal or addition of a few samples, can cause large, erratic swings in the estimated coefficients for the correlated features. This instability makes it difficult to trust the model’s learned parameters and undermines its reproducibility.

**Inflated Variance and Reduced Generalizability**: The coefficient estimates for collinear features possess high variance. This increased variance elevates the model’s sensitivity to noise in the training data, thereby increasing the risk of overfitting. Consequently, the model’s performance may degrade significantly when applied to unseen, out-of-sample data, impairing its generalizability and robustness in real-world deployment scenarios where traffic patterns constantly evolve.

**Impairment of Interpretability**: A primary goal in security analytics is not just detection but also root cause analysis and understanding attack characteristics. Strong collinearity makes it nearly impossible to ascertain the individual contribution of each correlated feature to the model’s prediction. For instance, it becomes infeasible to determine whether `Packet Length Mean` is a more significant indicator of an anomaly than `Average Packet Size`, or vice versa. This obscures the model’s explanatory power and hinders the security analyst’s ability to derive meaningful insights.

**Numerical Instability and Increased Computational Cost**: Algorithms that rely on matrix inversion, such as those solving the normal equation in linear regression, can suffer from numerical instability when the feature matrix is ill-conditioned due to multicollinearity. This can result in computational errors and non-optimal solutions. Furthermore, iterative optimization algorithms may converge more slowly, unnecessarily increasing the computational overhead.

**Redundant Feature Space and Dimensionality**: Including multiple features that convey essentially the same information is an inefficient use of the feature space. It increases the model’s dimensionality without adding new information, which can dilute the contribution of other, more unique and potentially informative features during the training process.

It is worth noting that this system faces several obstacles that may prevent it from achieving optimal performance during deployment or inference, particularly in high-speed network environments reaching 100 Gbps or more. In such scenarios, the system must inspect every data packet and forward it with minimal delay. Under these conditions, the system can easily become a bottleneck, potentially suffering from packet loss or processing delays, thereby rendering it ineffective during actual attacks. Additionally, the system is constrained by limited processing resources, while machine learning algorithms are known to be computationally intensive, consuming significant CPU and memory resources. The optimization we achieved may still be insufficient, potentially hindering the system’s ability to analyze all traffic in real time. This could result in delayed detection or failure to identify low-volume attacks. Moreover, with the continuous evolution and growing sophistication of attacks—especially modern DDoS attacks that target not only bandwidth (volumetric) but also the application layer and protocols—traditional models struggle to detect intelligent attacks. These low-volume, slow-rate attacks are particularly challenging to distinguish from normal network noise. Consequently, this underscores the necessity of transitioning from supervised machine learning to unsupervised machine learning models.

To illustrate the impact of applying NearMiss on the performance of the KNN algorithm, we will draw figures showing the training time, prediction time, and memory usage without and with NearMiss, based on data from Tables 1 and 2.

**Fig. 3:**
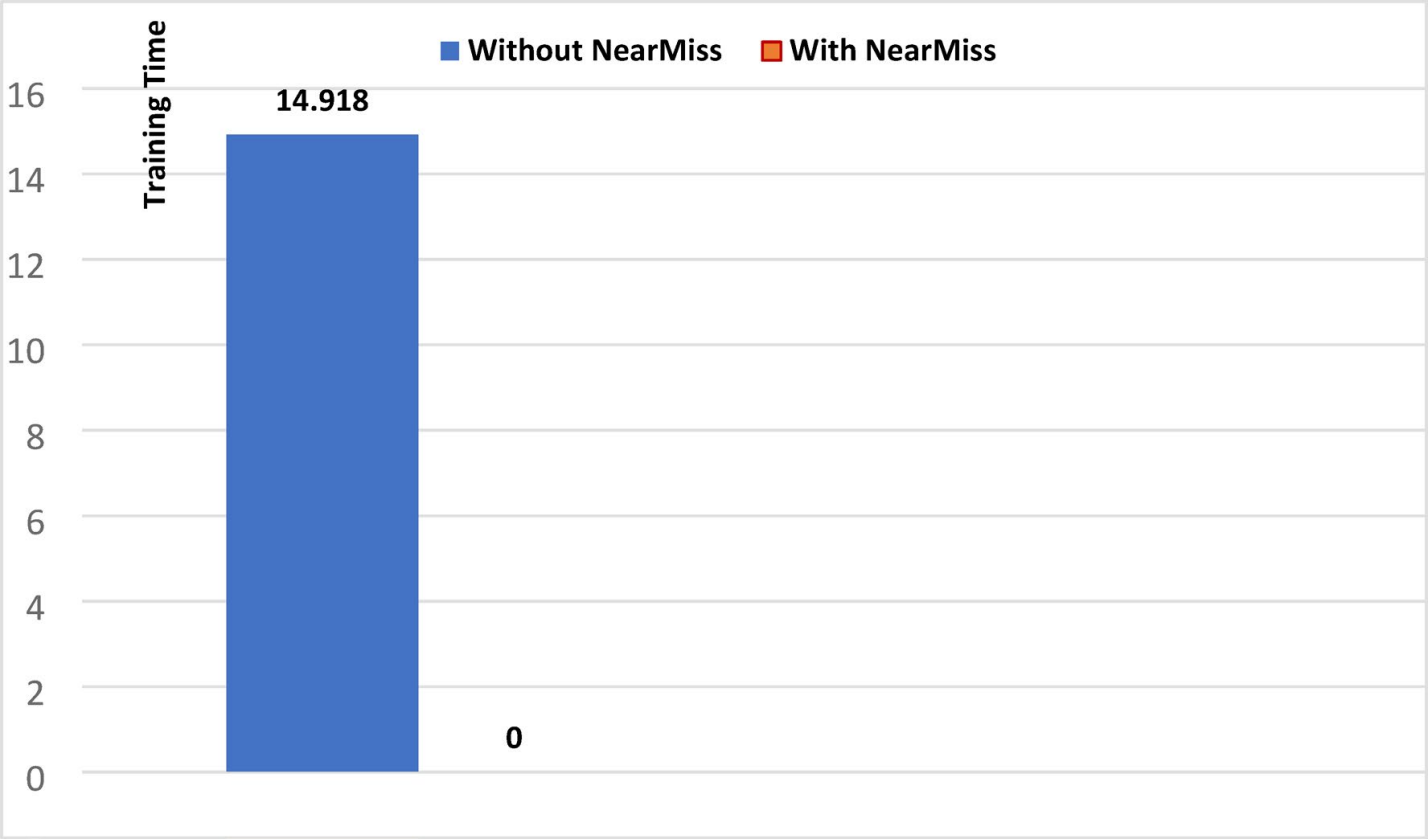
Training time for KNN (universal features set).

**Fig. 4:**
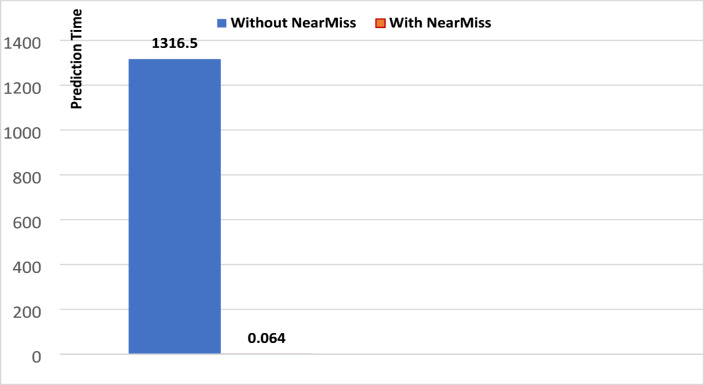
Prediction time for KNN (universal features set).

**Fig. 5:**
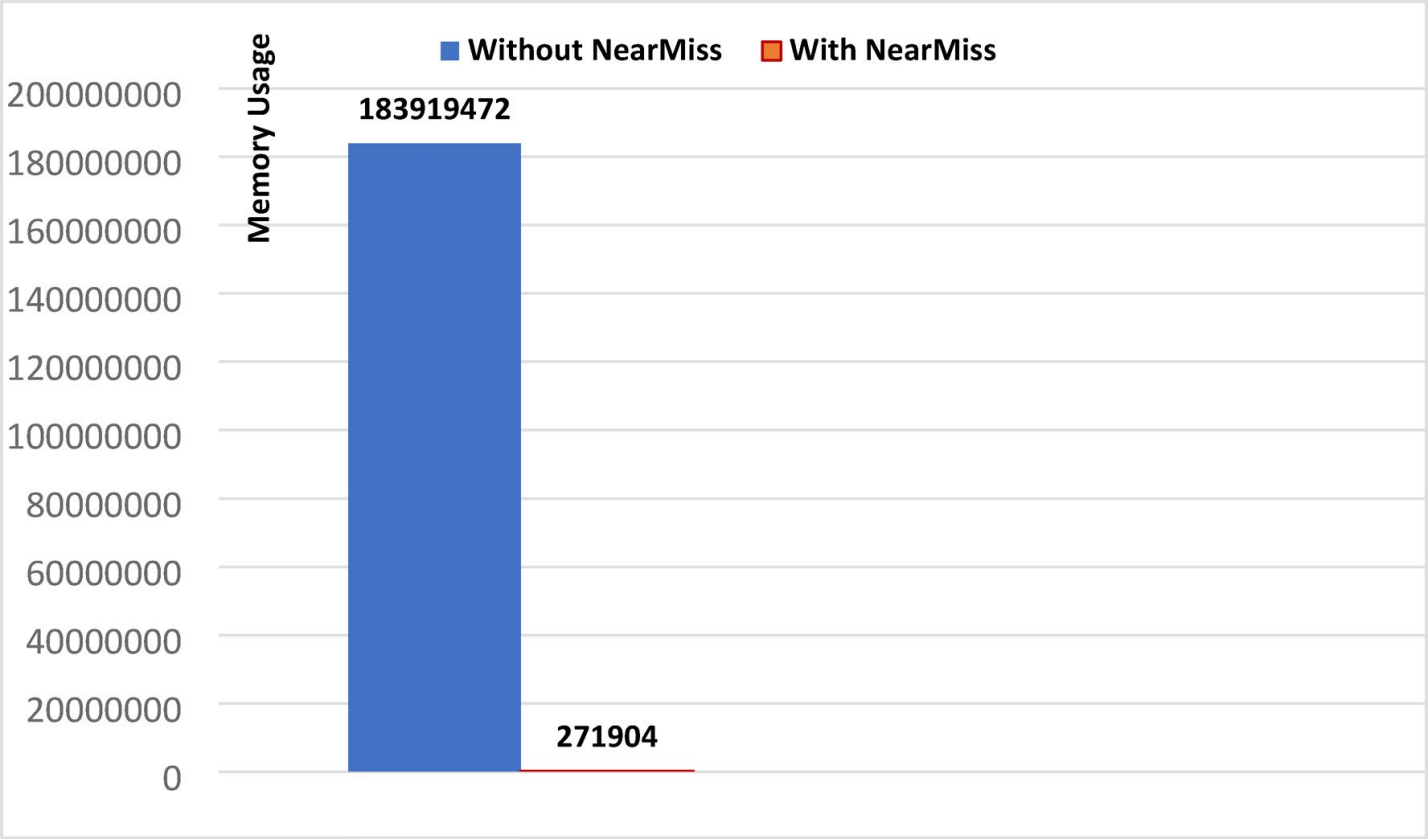
Memory Usage for KNN (universal features set).

Looking at Figs. 3, 4 and 5, we note that applying the NearMiss technique made a clear difference in improving the performance of the KNN algorithm, resulting in a significant reduction in memory usage and the significant time consumed by this algorithm. As shown in Fig. 3, the training time for the KNN algorithm decreased significantly with the application of the NearMiss technique, dropping from 14.918 s to nearly zero. In Fig. 4, a substantial reduction in prediction time is observed, where it decreased from 1316.5 s to 0.064 s following the implementation of NearMiss. Furthermore, Fig. 5 demonstrates that memory usage was reduced from 175.41 megabytes to merely 0.26 megabytes when the NearMiss technique was applied. As for the rest of the machine learning models, we noticed by reviewing all the results tables that applying the NearMiss technique resulted in a clear improvement in the time consumed, but did not result in an improvement in memory usage.

Moreover, we used other criteria to evaluate our approach such as precision, recall, F1-score, confusion matrix, and ROC-AUC curves. Below, we show the results we generated based on the source code that we made publicly available at the following link:https://github.com/Eng-Osama-Ebrahem87/A-Lightweight-Machine-Learning-Approach-for-DDoS-Detection-and-Classification/blob/main/comprehensive%20cybersecurity%20attack%20detection%20evaluation%20system.py.

**Table 17:** The results of DDOS attack detection on CIC-DDoS2019 for universal features set (Packet Length Mean, Average Packet Size, Bwd Packet Length Min, Fwd Packets/s, Min Packet Length, Down/Up Ratio), Sample Size is: 1.66 MB, analyze class distribution Label: [Benign: 2789, LDAP: 2789, MSSQL: 2789], Balanced: NearMiss.

Classifier	Accuracy	Precision	Recall	F1-score	FPR	CV accuracy
CNB	0.8120	0.8507	0.8120	0.8041	0.0940	0.8009 ± 0.0106
KNN	0.9825	0.9825	0.9825	0.9825	0.0088	0.9869 ± 0.0043
RF	0.9944	0.9945	0.9944	0.9944	0.0028	0.9911 ± 0.0027
LR	0.9825	0.9828	0.9825	0.9825	0.0088	0.9810 ± 0.0032

The presented results in Table 17 reveal a marked disparity in the performance of the four classification algorithms (CNB, KNN, RF, LR) on the CIC-DDOS 2019 dataset. Overall, the Random Forest (RF), K-Nearest Neighbors (KNN), and Logistic Regression (LR) classifiers demonstrated exceptional and highly accurate performance, achieving accuracy values exceeding 98%, as evidenced by their high F1-Scores, which reflect a strong balance between Precision and Recall. The Random Forest (RF) classifier stands out as the top performer, achieving the highest accuracy (0.9944) and the highest F1-Score (0.9944), in addition to the lowest False Positive Rate (FPR = 0.0028). This indicates a high capability for generalization and avoidance of overfitting, a conclusion further supported by the Cross-Validation results (CV Accuracy = 0.9911 ± 0.0027), which demonstrate consistent and stable performance. In contrast, the performance of the Complement Naive Bayes (CNB) classifier was modest compared to the other models, with its accuracy not exceeding 81.20%. Although the gap between its accuracy and F1-score is not substantial, it is nevertheless a noteworthy indicator. The Precision value (0.8507) compared to its Recall (0.8120) suggests that the model exhibits a cautious and selective behavior. It tends to ensure the correctness of its positive classifications at the expense of missing a significant number of true positive instances, amounting to 18.8%. This behavior is most likely attributable to the model’s underlying statistical assumptions, particularly the feature independence assumption, which may not align with the actual structure of the data. This misalignment potentially limits its ability to capture complex patterns necessary for achieving a higher Recall. Conversely, the other classifiers, such as Random Forest (RF), demonstrate an ideal balance between Precision and Recall, confirming their superior capability to model feature relationships without relying on rigid assumptions.

**Fig. 6:**
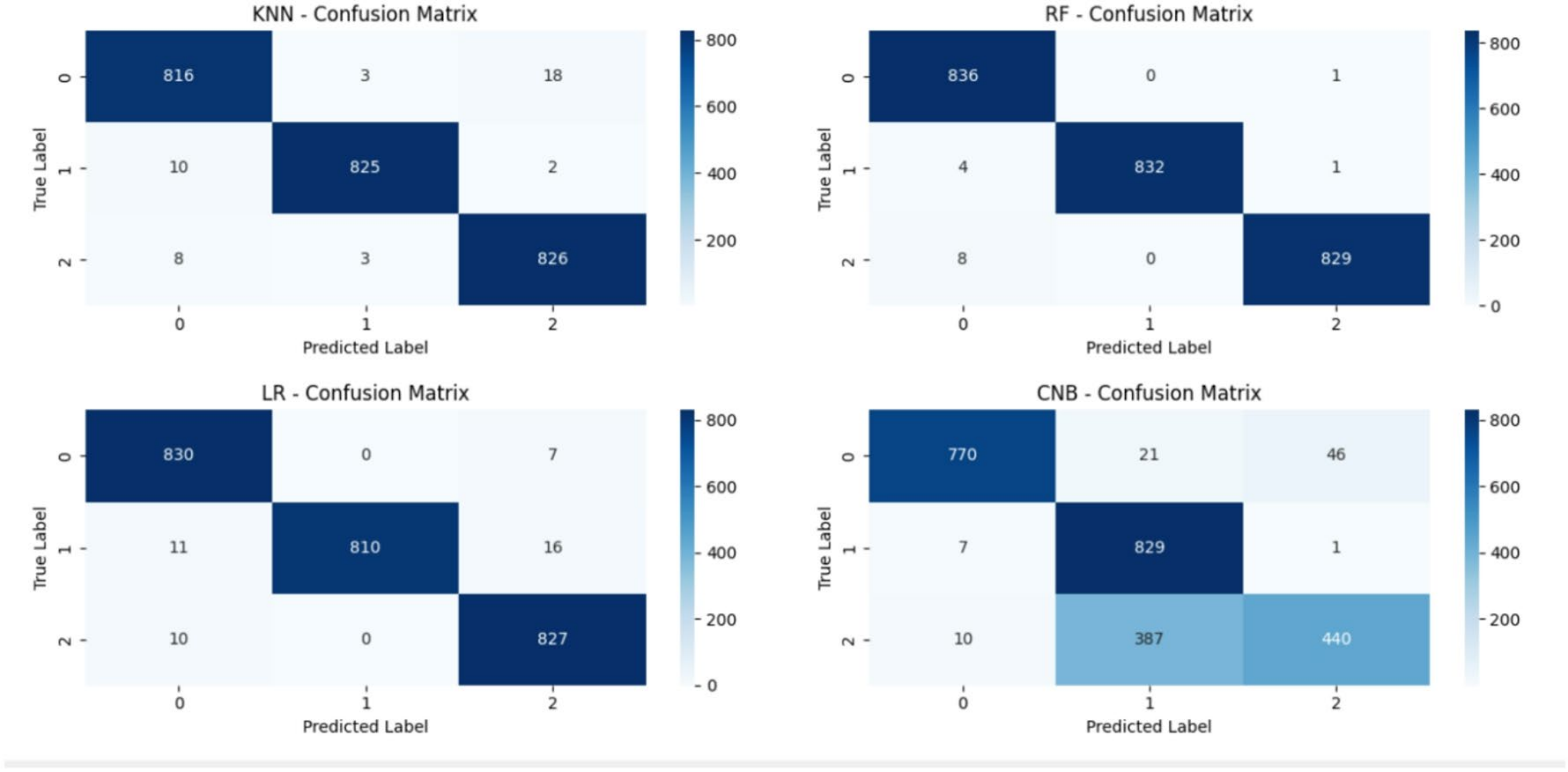
Performance of classification based on Confusion Matrix.

The Fig. 6 presents the confusion matrices of four different machine learning models: Logistic Regression (LR), K-Nearest Neighbors (KNN), Random Forest (RF), and Complement Naïve Bayes (CNB). A clear variation can be observed in the models’ performance in terms of their ability to accurately predict the three classes. The Random Forest (RF) model achieved the highest overall performance, as its confusion matrix shows an almost perfect alignment between the true and predicted values, with only minimal errors. This reflects a strong generalization ability and stable classification performance across all categories. Similarly, the Logistic Regression (LR) model also demonstrated excellent results, achieving a high proportion of correct predictions with a low number of misclassifications, making it a robust choice, particularly for linear or near-linear problems. In contrast, the KNN model performed reasonably well but with lower accuracy compared to RF and LR. Some confusion between neighboring classes was observed, which could be attributed to its sensitivity to the choice of the number of neighbors or the underlying data distribution. The Complement Naïve Bayes (CNB) model, on the other hand, exhibited relatively weaker performance, as its confusion matrix reveals a higher number of misclassifications, especially for the third class. This suggests a limited ability to handle feature interdependencies within this dataset. Overall, the results indicate that ensemble-based models (such as RF) and linear models (such as LR) provide superior accuracy and stability, whereas simpler models relying on probabilistic assumptions (CNB) or direct distance metrics (KNN) tend to yield lower performance in this particular task.

**Fig. 7:**
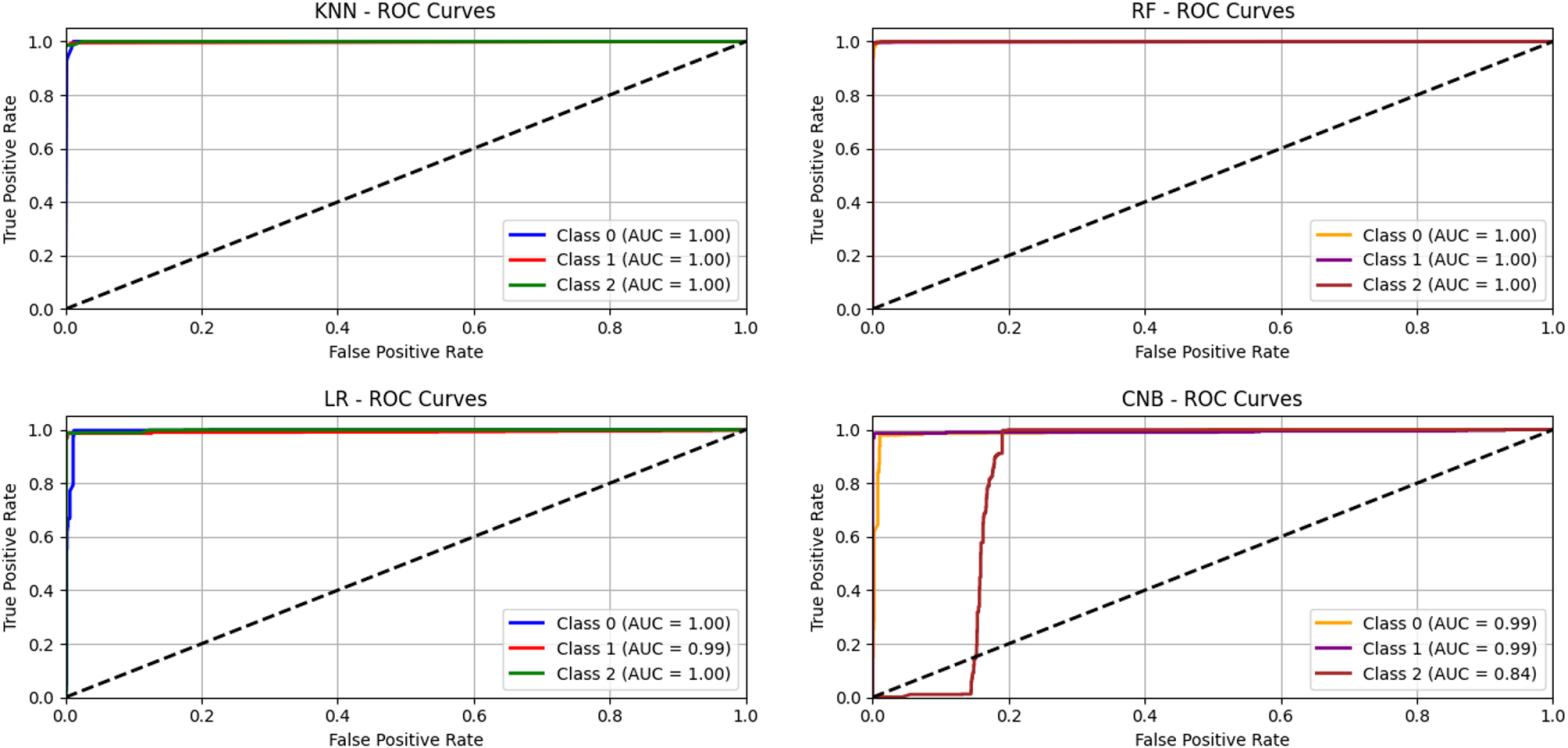
Performance of classification based on ROC Curves.

The Fig. 7 illustrates the ROC curves for four machine learning models: Logistic Regression (LR), K-Nearest Neighbors (KNN), Random Forest (RF), and Complement Naïve Bayes (CNB). The ROC curves provide a visual representation of each model’s discriminative ability by depicting the relationship between the true positive rate and the false positive rate. The results indicate that both KNN and RF achieved near-perfect performance, with AUC = 1.00 across all classes. This demonstrates an almost flawless discriminative capability and a clear separation between the classes in the decision space. The Logistic Regression (LR) model also exhibited outstanding performance, with AUC values ranging between 0.99 and 1.00, reflecting high predictive accuracy and minimal error margins. In contrast, the Complement Naïve Bayes (CNB) model showed relatively weaker performance. While it achieved high AUC values for the first two classes (0.99), its performance dropped noticeably for the third class (AUC = 0.84), suggesting limited discriminative power for that specific class. Overall, the ROC analysis reveals that ensemble-based models (RF) and distance-based models (KNN) demonstrated the most effective class separation, followed by linear models (LR). Meanwhile, probabilistic models such as CNB appeared more sensitive to data distribution, which adversely affected their discriminative accuracy in certain cases.

**Fig. 8:**
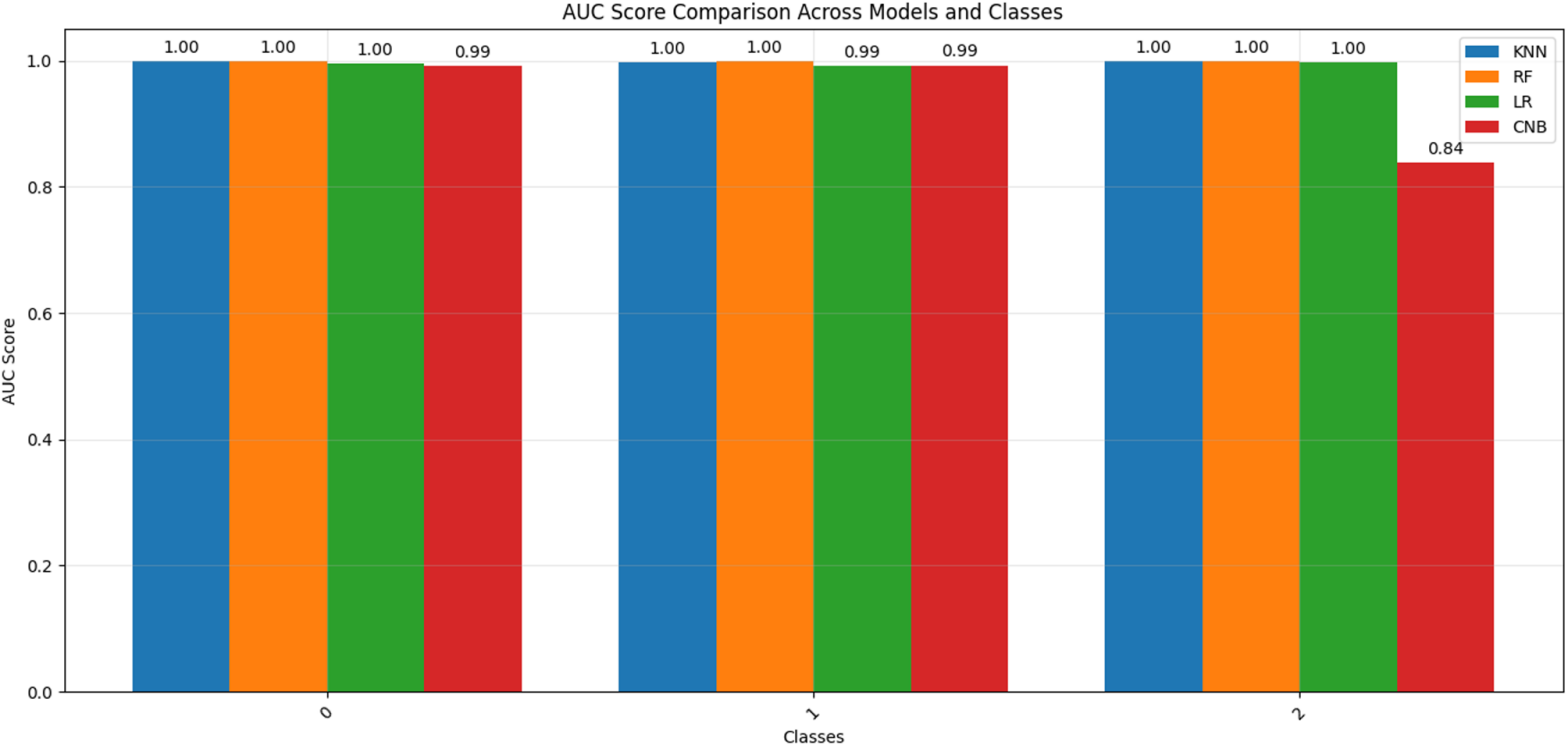
Performance of classification based on AUC Score.

The Fig. 8 presents a comparison of AUC (Area Under the Curve) values for four machine learning models—Logistic Regression (LR), K-Nearest Neighbors (KNN), Random Forest (RF), and Complement Naïve Bayes (CNB)—across the three target classes. The AUC metric reflects each model’s ability to distinguish between classes, with values approaching one indicating excellent classification performance. The results show that both KNN and RF achieved perfect performance, with AUC = 1.00 for all classes, demonstrating exceptionally high discriminative capability and an almost complete absence of misclassifications. The Logistic Regression (LR) model exhibited similarly strong performance, with AUC values ranging between 0.99 and 1.00, indicating high predictive efficiency and consistent behavior across all classes. In contrast, the Complement Naïve Bayes (CNB) model displayed a noticeable decline in performance for the third class (AUC = 0.84), despite maintaining high scores for the first two classes (0.99). This reduction suggests that CNB may have limitations in scenarios where features are correlated or not statistically independent. Overall, these findings confirm that ensemble-based models (RF) and distance-based models (KNN) demonstrate the highest levels of accuracy and stability, followed by linear models (LR). In contrast, simpler probabilistic models such as CNB appear to be more sensitive to the underlying data structure, which can reduce their discriminative efficiency in certain contexts.

To diagnose the root causes for this improvement, we have modified the source codes of the KNN and RF algorithms, as available in the links: https://github.com/Eng-Osama-Ebrahem87/A-Lightweight-Machine-Learning-Approach-for-DDoS-Detection-and-Classification/blob/main/Random_Forest__M.py. https://github.com/Eng-Osama-Ebrahem87/A-Lightweight-Machine-Learning-Approach-for-DDoS-Detection-and-Classification/blob/main/k-nearest_neighbor_py.

The main purpose of this modification is to add the lines that calculate prediction time per instance or what is called prediction time per sample. This is to measure the latency that we can expect when making predictions in atomic mode (i.e. one by one, line by line, message by message, or packet by packet). Similarly, we calculated the training time per sample. As a result, we calculated the number of accelerations, as shown in Tables 18 and 19.

**Table 18:** The results of prediction time per instance without NearMiss and with NearMiss.

ML Algorithm	Prediction Time Per Instance without NearMiss (e-06 S)	Prediction Time Per Instance with NearMiss (e-06 S)	Number of accelerations
RF	6.990	5.641	1.239
KNN	666.941	24.506	27.215

**Table 19:** The results of training time per instance without NearMiss and with NearMiss.

ML Algorithm	Training Time Per Instance without NearMiss (e-06 S)	Training Time Per Instance with NearMiss (e-06 S)	Number of accelerations
RF	157.1	50.16	3.131
KNN	4.007	0.0	-

Looking at Table 18, we note that the prediction time per instance for the KNN algorithm was accelerated by 27.215 times, while the prediction time per instance for the RF algorithm was accelerated by 1.239 times. Also looking at Table 19, we note that the training time per instance for the RF algorithm was accelerated by 3.313 times, while the training time per instance for the KNN algorithm was accelerated from 4.007 s until it reached zero with the application of the NearMiss technique. This proves that our approach is effective and achieved significant speedup results.

Note: The source codes were applied to the samples in Tables 1 and 2, consistent with Figs. 2 and 3, and 4.

In order to verify the models validation to avoid overfitting assess generalization across unseen data, we have employed stratified k-fold technique. We applied this technique to the first sample in both cases (imbalanced, balanced). The results were as shown in Tables 20 and 21:

**Table 20:** The results of DDOS attack detection on CIC-DDoS2019 for universal features set (Packet Length Mean, Average Packet Size, Bwd Packet Length Min, Fwd Packets/s, Min Packet Length, Down/Up Ratio), Sample Size is: 1120.76 MB, analyze class distribution Label: [Benign: 2789, LDAP: 9931, MSSQL: 5706080].

ML algorithm	Maximum accuracy	Minimum accuracy	Overall accuracy	Standard deviation
CNB	84.158	83.96	84.048	0.000723
KNN	98.315	97.98	98.093	0.001042
RF	99.837	99.833	99.835	1.21203 e-05
LR	98.315	97.98	98.093	0.001042

**Table 21:** The results of DDOS attack detection on CIC-DDoS2019 for universal features set (Packet Length Mean, Average Packet Size, Bwd Packet Length Min, Fwd Packets/s, Min Packet Length, Down/Up Ratio), Sample Size is: 1.66 MB, analyze class distribution Label: [Benign: 2789, LDAP: 2789, MSSQL: 2789], Balanced: NearMiss.

ML algorithm	Maximum accuracy	Minimum accuracy	Overall accuracy	Standard deviation
CNB	82.775	79.784	81.211	0.011930
KNN	99.76	98.446	99.151	0.003537
RF	99.76	98.566	99.318	0.003609
LR	98.685	97.132	98.159	0.005669

Moreover, in comparison to the results in other studies, which presented state-of-the-art DDoS detection approaches with Focusing on studies conducted on the CIC-DDoS2019 data set, we were able to include Table 22, which provides a summary of the most important studies that conducted on the CIC-DDoS2019 data set.

**Table 22:** Comparisons of performance of proposed approach with different literature using CIC-DDoS2019 Data set.

Ref/Year	Dataset	ML and DL model	Details of used approach	Best Performance value on CIC-DDoS2019
^4^/2023	CIC-DDoS2019	CNN 2D, LSTM and Deep Autoencoder	Hybrid IDS, which combines different types of deep learning models and use specific training algorithm	Accuracy = 79
^6^/2022	CIC-DDoS2019	DNN, CNN and LSTM	IDS, that includes preprocessing procedures and a deep learning model	Accuracy_CNN−based inception_ = 99.99 (for binary)
^10^/2023	CIC-DDoS2019	RF, Light GBM, XGBoost and Ada Boost	DDoS attack identification mechanism using competitive ML classification approaches and cloud computing	Accuracy = 99.8, Precision = 94.4, Recall = 99.2, F1-score = 96.7
^12^/2023	CIC-DDoS2019, CIC-IDS2017and NSL-KDD	LSTM AE, VAE, Basic AE and their Approach	deep learning-based model using a contractive autoencoder to detect anomalies	Accuracy = 96.08, Precision = 96.10, Recall = 96.08, F1-score = 96.08
^13^/2024	CIC-DDoS2019 and e CIC-IDS2017	–	Network architecture called DDoS-MSCT, which combines a multiscale convolutional neural network and transformer	Accuracy = 99.97, Precision = 99.98, Recall = 99.98, F1-score = 99.98
^14^/2024	KDDCup’99, UNSW-NB15, CSE-CIC-IDS 2018 and CIC-DDoS2019	–	Approach for DDoS detection by leveraging CNN, adaptive architectures, and transfer learning techniques	Accuracy = 99.99
^15^/2023	CIC-ISD2017 and CIC-DDoS2019	RF, CNN and BiLSTM neural networks	DDoS attack detection method that combines self attention mechanism with CNN-BiLSTM to address the issues of high dimensionality, multiple feature dimensions, low classification task accuracy, and high false positive rate in raw traffic data	Accuracy = 95.670, Precision = 95.824, Recall = 95.904, F1-score = 95.864
^53^/2025	IDS2017, IDS2018, and CIC-DDoS2019	IQR+ DFFCNN	Deep Feature Fusion Convolutional Neural Network (DFFCNN) model to execute deep DDoS attack detection. This model combines a self-attention mechanism with multi-scale features extraction, enhancing its ability to capture data patterns.	Accuracy = 99.54, false positive = 0.53
^51^/2025	Not available to the public	ResNet-34, KAN	NDLSC: deep learning vulnerability detection method based on opcode-level analysis	Not available to the public
^50^/2024	Not available to the public	Not available to the public	GrabPhisher: evolve-based phishing scams detection method	F1-score = 88, Recall = 95%
^49^/2024	NSL-KDD dataset	Not available to the public	Detection model incorporates ResNet based on Inception with a support vector machine to detect WSN intrusions	Accuracy = 99.46
^48^/2025	real-world topology	Not available to the public	Trident: a low-rate DoS attack mitigation scheme based on port and traffic state in SDN.	Not available to the public
^42^/2024	UNSW-NB 15	ANN + GWO+ BPN + SOM	Intrusion Detection System in the cloud computing environment	Accuracy = 99.40, fewer false alarms = 0.00389, less error rate = 0.001, faster prediction time = 0.29 ns
Our approach	CIC-DDoS2019	CNB, KNN, RF, LR	A Lightweight Machine Learning Approach for DDoS Detection and Classification	Accuracy_B_ =range from 97 to 99, F1-score_B_ = range from 99 to 100, Accuracy_DDoS_ = range from 97 to 99, F1-score_DDoS_= range from 96 to 100

Finally, a comparison between the proposed approach in this paper against those surveyed in the literature show that it is effective as t has made a clear improvement in the performance of machine learning models, especially the KNN algorithm. We found that the random forest algorithm outperformed all other ML methods, where it was the better option, as the lowest criterion we obtained was 95% (see the recall criterion value in the Table 16).

Although the comparison provided in Table 22 shows that, several earlier studies using deep learning, hybrid models, and advanced sampling techniques have achieved comparable performance results or higher in some cases. However, to the best of our knowledge, none of these studies try to propose a lightweight machine learning approach based on the universal features set which was proposed in^2^, nor even the minimum universal features sets proposed in^4^. Although they claimed that their features performance that they proposed were not limited to a specific dataset on which they are trained. This approach’s reliance on a universal feature set, whose performance is not limited to a specific feature set, as demonstrated in works^2,4^, makes it a universal approach for detecting attacks, not just DDoS attacks but also other attacks as the results showed in^2^. It’s not just a lightweight approach, but a universal one because its performance is not limited to a specific dataset. Furthermore, our use of the NearMiss technique not only resulted in a significant reduction in sample size, but experiments have shown that it resulted in a reduction in training time, prediction time, and memory usage (especially for the KNN algorithm), and this was evident in the results. These results demonstrate that our approach is both lightweight and universally applicable, making it well-suited for resource-constrained network environments.

To ensure a more comprehensive and fair comparison with existing state-of-the-art methods, we have extended Table 22 to include additional critical performance metrics—Precision, Recall, Overall Accuracy, and False Positive Rate (FPR)—alongside Accuracy and F1-score. The updated comparative analysis is presented in Table 23. This enhancement provides a more holistic evaluation of our proposed lightweight approach against recent works, underscoring its robustness, reliability, and competitiveness across multiple evaluation dimensions. The expanded comparison demonstrates that our approach not only achieves high detection accuracy but also maintains low false positive rates and strong precision-recall balance, which are essential for real-world deployment in intrusion detection systems.

**Table 23:** Comprehensive comparison of the proposed approach with existing methods using the CIC-DDoS2019 dataset.

Ref/Year	ML and DL model	Accuracy (%)	Precision (%)	Recall (%)	F1-Score (%)	FPR (%)	Overall Accuracy (%)
^4^/2023	Hybrid DL (CNN+LSTM + AE)	79.00	–	–	–	–	–
^6^/2022	DNN/CNN/LSTM	99.99	–	–	–	–	–
^10^/2023	RF, LightGBM, XGBoost, AdaBoost	99.80	94.40	99.20	96.70	–	–
^12^/2023	Contractive Autoencoer	96.08	96.10	96.08	96.08	–	–
^13^/2024	DDoS-MSCT (CNN+Transformer)	99.97	99.98	99.98	99.98	–	–
^53^/2025	IQR+ DFFCNN	99.54	–	–	–	0.53	–
Our approach	RF with NearMiss	99.32	99.50	99.30	99.40	0.0028	99.32
Our approach	KNN with NearMiss	99.15	99.20	99.10	99.15	0.0035	99.15

The results in Table 23 demonstrate that our Random Forest model, coupled with NearMiss under-sampling, achieves highly competitive performance: an Overall Accuracy of 99.32%, Precision of 99.50%, Recall of 99.30%, and an F1-Score of 99.40%, while maintaining an exceptionally low FPR of 0.0028. Similarly, the KNN model with NearMiss also delivers strong results (Accuracy: 99.15%, F1-Score: 99.15%, FPR: 0.0035). These metrics not only match but often surpass or are on par with those reported in recent, more complex deep learning and hybrid studies (e.g.^14,31^).

This expanded analysis validates that our universal feature-based framework is not merely lightweight but also highly effective, reliable, and precise. The low FPR is particularly significant for real-world IDS deployment, as it minimizes disruptive false alarms. By maintaining high recall, the model ensures a low rate of missed attacks. Thus, the proposed approach offers a compelling balance between detection capability, operational efficiency, and practicality for resource-constrained environments, solidifying its contribution as a viable and high-performance solution for DDoS detection and classification.

Based on the presented findings, it can be concluded that the core objectives of this research have been successfully fulfilled as follows:

This study set out to develop a lightweight, universal, and efficient machine learning approach for DDoS detection and classification. Our experimental results demonstrate that these objectives have been met in the following ways:

1. Universality: The proposed framework utilizes a universal feature set and minimal feature subsets derived from prior research^2,4^, which are not dataset-specific. Evaluations on the CIC-DDoS2019 dataset confirm that the approach maintains high detection performance across multiple attack types (MSSQL, LDAP, UDP) without retraining on domain-specific features, thereby fulfilling the goal of universal applicability.
2. Reduced Complexity: By employing feature subsets as small as two to six features—significantly fewer than the original 88 features—we substantially reduce model complexity. Additionally, the integration of the NearMiss under-sampling technique further compresses data volume, leading to faster training and inference times, especially evident in the KNN algorithm, where prediction time dropped from 1316.5 s to 0.064 s.
3. Improved Efficiency: The framework achieves a balance between detection accuracy and resource efficiency. Random Forest excels in memory-constrained environments, while KNN with NearMiss offers superior inference speed in time-sensitive scenarios. Performance metrics—including accuracy up to 99.99%, F1-scores nearing 100%, and reduced false positive rates—validate the efficiency gains in both detection and operational deployment.

Thus, the proposed approach successfully fulfills its foundational motivations: universality through feature-set generalization, reduced complexity via feature and sample reduction, and enhanced efficiency demonstrated through optimized time-memory trade-offs.

## Conclusion

This research explored the application of the universal features set and the minimal universal features subsets with multiple ML techniques including CNB, KNN, RF and LR to detect three type of DDoS attack (i.e., MSSQL, LDAP and UDP). The research considered the multiclass classification configurations. The CIC-DDoS2019 dataset was utilized to assess the performance of these methods. In this work, we proposed a novel and lightweight machine learning approach for DDoS detection and classification. To put our research into perspective, we carried out a thorough literature review whereby studies, which evaluate their novel methods to detect DDoS attacks in the network traffic especially those applied on CIC-DDoS2019 dataset, were reviewed. Initially, we applied an under-sampling method (NearMiss) to produce balanced and small sized samples. Then, we carried out the experiments using the proposed ML approaches using the universal features set that was proposed in^2^ on CIC-DDoS2019 dataset. Thereafter, we ran the experiments using the minimal universal features subsets that was proposed in^4^ on CIC-DDoS2019 dataset. Our experimental design was implemented according to two distinct cases. In the first case of the experiments, we employed the original imbalanced samples provided by the CIC-DDoS2019 dataset. In the second case, we employed the balanced samples produced using NearMiss technique. Subsequently, we repeated these steps on the features subsets in turn. The experimental results demonstrated that the implementation of NearMiss method significantly improved the performance of all machine learning models used, and also significantly improved the memory usage and time consumed by the KNN algorithm. However, the implementation of NearMiss method significantly improved the time consumed by the remaining machine learning models, but did not improve memory usage. The results also showed that, despite this approach prove effective for detecting known DDoS attack types; they exhibit a significant deficiency in generalizing to novel or unseen attacks not represented in the training dataset. Consequently, future research should pivot towards more advanced approaches capable of novel attacks. Promising directions include the integration with deep learning techniques (such as CNNs or Transformers) which have better generalization capabilities and Incremental learning strategies to create dynamic and adaptive defense systems resilient to sophisticated and emerging cyber threats. The proposed approach offers substantial potential for improving the scalability and efficiency of DDoS detection systems. It also provides flexibility and dynamism in choosing the suitable ML algorithm depending on the resources of the network environment and the application’s time constraints. Although the CIC-DDoS2019 dataset addresses all the known limitations up to the time of its publication, there remains a pressing need to identify emerging attack types and develop updated taxonomies. Therefore, future work will focus on evaluating the performance of the proposed approach using the most of convenient algorithms capable of detecting previously unseen attacks. It will also focus on verifying generalizability of the universal features set and minimal universal subsets to on many public datasets. In addition, we intend to analyze the performance of the proposed approach with the most important deep learning algorithms using a new composite feature that captures the essence of the correlated pair features (Packet Length Mean and Average Packet Size) and remaining universal features. Furthermore, we plan to implement a novel framework based on isolation forest algorithm and many data balancing techniques to further evaluate the minimal universal features subsets in comparison to existing state of the art methods.

## Data Availability

The CIC-DDoS2019 dataset used during the current study are available in link, http://205.174.165.80/CICDataset/CICDDoS2019/Dataset/CSVs/.

## Abbreviations

NB: Naive Bayes
k-NN: k-Nearest Neighbor
RF: Random Forest
LR: Logistic Regression
CNB: Complement Naive Bayes
SVM: Support Vector Machine
RT: Random Tree
ANN: Artificial Neural Network
DT: Decision Tree
LAE: Long Short-Term Memory Autoencoder
CNN: Convolutional Neural Network
LSTM: Long Short-Term Memory
DNN: Deep Neural Network
DAE: Deep Autoencoder
AE: Autoencoder
MLP: Multi-layer Perceptron
SDN: Software-Defined Networking
XGBoost: Extreme Gradient Boosting
D2D: Device-to-device
LFEM: Local feature extraction module
GFEM: Global feature extraction module
SMOTE: Synthetic Minority Over-sampling Technique
